# Vancomycin and nisin-modified magnetic Fe_3_O_4_@SiO_2_ nanostructures coated with chitosan to enhance antibacterial efficiency against methicillin resistant *Staphylococcus aureus *(MRSA) infection in a murine superficial wound model

**DOI:** 10.1186/s13065-024-01129-y

**Published:** 2024-02-23

**Authors:** Mona Nasaj, Abbas Farmany, Leili Shokoohizadeh, Farid Aziz Jalilian, Reza Mahjoub, Ghodratollah Roshanaei, Alireza Nourian, Omid Heydari Shayesteh, Mohammadreza Arabestani

**Affiliations:** 1 Department of Microbiology, Faculty of Medicine, Hamadan University of Medical Sciences, Hamadan, Islamic Republic of Iran; 2 Dental Research Center, School of Dentistry, Hamadan University of Medical Sciences, Hamadan, Islamic Republic of Iran; 3 Department of Virology, Faculty of Medicine, Hamadan University of Medical Sciences, Shahid Fahmideh Street, Park Mardome, Hamadan, Islamic Republic of Iran; 4 Department of Pharmacology and Toxicology, School of Pharmacy, Medicinal Plants and Natural Products Research Center, Hamadan University of Medical Sciences, Hamadan, Islamic Republic of Iran; 5 Department of Biostatistics, School of Health, Hamadan University of Medical Sciences, Shahid Fahmideh Street, Park Mardome, Hamadan, Islamic Republic of Iran; 6 Department of Pathobiology, School of Veterinary Science, Bu-Ali Sina University, Hamedan, Islamic Republic of Iran; 7 Department of Medicinal Chemistry, School of Pharmacy, Hamadan University of Medical Sciences, Hamadan, Islamic Republic of Iran; 8 Infectious Disease Research Center, Hamadan University of Medical Sciences, Hamadan, Islamic Republic of Iran

**Keywords:** Chitosan-coated magnetic nanoparticle, Nisin, Vancomycin, MRSA, Antibacterial activity, Conjugation efficacy

## Abstract

**Background:**

The objective of this research was to prepare some Fe_3_O_4_@SiO_2_@Chitosan (CS) magnetic nanocomposites coupled with nisin, and vancomycin to evaluate their antibacterial efficacy under both in vitro and in vivo against the methicillin-resistant *Staphylococcus. aureus* (MRSA).

**Methods:**

In this survey, the Fe_3_O_4_@SiO_2_ magnetic nanoparticles (MNPs) were constructed as a core and covered the surface of MNPs via crosslinking CS by glutaraldehyde as a shell, then functionalized with vancomycin and nisin to enhance the inhibitory effects of nanoparticles (NPs). X-ray diffraction (XRD), Fourier-transform infrared spectroscopy (FT-IR), field emission scanning electron microscope (FE-SEM), vibrating sample magnetometer (VSM), and dynamic light scattering (DLS) techniques were then used to describe the nanostructures.

**Results:**

Based on the XRD, and FE-SEM findings, the average size of the modified magnetic nanomaterials were estimated to be around 22–35 nm, and 34–47 nm, respectively. The vancomycin was conjugated in three polymer-drug ratios; 1:1, 2:1 and 3:1, with the percentages of 45.52%, 35.68%, and 24.4%, respectively. The polymer/drug ratio of 1:1 exhibited the slowest release rate of vancomycin from the Fe_3_O_4_@SiO_2_@CS-VANCO nanocomposites during 24 h, which was selected to examine their antimicrobial effects under in vivo conditions. The nisin was grafted onto the nanocomposites at around 73.2–87.2%. All the compounds resulted in a marked reduction in the bacterial burden (P-value < 0.05).

**Conclusion:**

The vancomycin-functionalized nanocomposites exhibited to be more efficient in eradicating the bacterial cells both in vitro and in vivo. These findings introduce a novel bacteriocin–metallic nanocomposite that can suppress the normal bacterial function on demand for the treatment of MRSA skin infections.

**Supplementary Information:**

The online version supplementary material available at 10.1186/s13065-024-01129-y.

## Background

*Staphylococcus aureus* (*S. aureus*) infections, in particular *S. aureus* bacteremia (SAB) outbreaks, are of great importance in the areas of both antimicrobial resistance (AMR) and healthcare-associated infections (HAI). Methicillin-resistant S. aureus (MRSA) is an emerging pathogen of major concern in relation to antimicrobial resistance (AMR), according to the World Health Organization [1]. The Centers for Disease Control and Prevention (CDC) have revealed that at least 2.8 million people are affected by antibiotic-resistant illnesses, which have been linked to over 35,000 deaths [2]. According to a British survey on antimicrobial resistance (AMR), around 700,000 people are dying every year in the world because of the antibiotic-resistant diseases [3]. Through a series of sequential genetic mutations and the horizontal transfer of mobile genetic elements, multidrug-resistant (MDR) bacteria can acquire the resistance that renders most antibiotics ineffective, including those that are considered a last resort. This underscores the need for the development of more effective antimicrobial agents [4]. Furthermore, the conventional antimicrobials indiscriminately eradicate beneficial microorganisms and detrimentally disrupt the commensal human microbiota [5]. This emphasizes the necessity to develop the novel strategies that employ distinct bactericidal pathways to overcome drug resistance, targeting only harmful bacteria with negligible impact on the patients and all other beneficial microbes [6]. In addition, biofilm-related antimicrobial resistance is a further cause for concern. Therefore, the nanocarriers seem to have a role in overcoming these barriers to improve effectiveness, as they serve as a protective layer that shields against interactions and limits inactivation of drugs by biofilm and topical enzymes. In this case, drug delivery nanosystems hold significant promise for treating infections brought on by intracellular or biofilm-forming substances [7]. Based on the inorganic forms of nanoparticles (NPs), nanomaterial carriers typically comprise an organic coating (substances based on carbon) and an inorganic core (structures based on metal or oxide) that support the conjugation of biomolecules and/or serve as shields to prevent the inner core from unfavorable physicochemical reactions with the biological compartments [8, 9]. Carbon nanostructures like polymers, dendrimers, exosomes, micelles, liposomes, and solid lipid NPs are among organics nanomaterials [10, 11]. While the majority of inorganic materials have a relatively smaller particle size, superior stability, controlled tunability, enhanced permeability of the encapsulated drug, the ability to increase drug-loading efficiency, and the ability to release the drugs over extended periods of time, the majority of organic nanoparticles composed of biodegradable materials are biocompatible and nontoxic [12–14]. Chitosan (CS), and chitin are recognized as the most versatile biopolymers occurring affluently in nature. CS obtained from the deacetylation of chitin, a major constituent of the crustacean exoskeleton [15, 16]. Because of its special qualities—such as biocompatibility, biodegradability, and non-toxicity—CS and CS-based nanocomposites are well-known. These properties have wide-ranging applications in a number of biomedical domains, including tissue engineering, drug delivery, and wound healing [17, 18]. Nisin is a member of the class of cationic peptide antimicrobials made from strains of *Lactococcus lactis* that are commonly used to preserve food [19]. It has been shown to work via synergistic pathways in combination with the traditional antibiotic therapies against MRSA and as an interfering agent in the formation of bacterial biofilm [20–22]. In the field of antibacterial agents, metal nanoparticles have attracted enormous interest due to they can be synthesized to have a large surface area and a high density of various reactive sites [23]. Due to their wide surface areas, intricate crystalline structures with many atoms positioned at edges and corners, and prevalence of additional potentially reactive sites, metal oxide nanoparticles behave more antimicrobially than metallic nanoparticles [24, 25]. The MNPs used for the biomedical purposes are usually composed of the elements iron and iron oxide with nanocrystalline magnetite (Fe_3_O_4_) cores because of their good biocompatibility, biodegradability, and facile synthesis [26]. Several scientists developed the Fe_3_O_4_-based nanocatalysts with an excellent catalytic activity that suggest a broad array of potential advantages, such as enhanced selectivity of the catalyst, broad surface area-to-volume ratio, improved reactivity, facile working (by an external magnet), a great reusability with no remarkable reduction in the catalytic performance, large extraction/or separation capability with high yields of products, reduced reaction durations, negligible amounts of wastes, excellent stability, the features that make magnetic-based nanocatalysts the promising alternative heterogeneous catalysts [27–33]. The challenge of removing the catalyst from the mixture and the impossibility of recycling it for use in additional processes are the drawbacks of homogeneous catalysis. Magnetic characteristics can be an important advantage for these materials, offering the possibility of their convenient manipulation and separation using an external magnetic force without taking prolonged times for filtration or centrifugation following completion of the reaction [28]. The CoFeO_2_O_4_@CS-based nanocomposites which have recently been developed due to their exceptional features, and thermal stability effects can be applied in the field of magneto-hyperthermia [34]. In addition, nano therapy systems based on FeO-NPs can help in the reduction of the therapeutic dosages of drugs to make them more effective and enable targeted drug delivery to the desired tissue or cell [26]. Fe_3_O_4_ magnetic nanoparticles tend to agglomerate and are highly prone to oxidization upon exposure to air [35]. Thus, it is necessary to consider surface modification with a wide range of available materials such as all kinds of polymeric materials, noble metals, silica, metal oxide, and graphene oxide materials [36, 37]. Due to its excellent durability against degradation, silica is still thought to be the best option for surface functionalization. Furthermore, because silica has a high concentration of silanol groups (–SiOH) on its surface, it enhances hydrophilicity, surface functionality, and biocompatibility, making it a highly promising material for use in a variety of biological disciplines [36, 38, 39]. In this study, we synthesized Fe_3_O_4_ supermagnetic nanoparticles functionalized with vancomycin, and nisin to enhance antibacterial performance against MRSA, ampicillin-resistant *S. aureus* (ARSA), and methicillin-susceptible *S. aureus* (MSSA) strains. Since nanoparticle agglomerates are highly prone to be removed from the bloodstream via reticuloendothelial system (RES) opsonization and phagocytosis [40], on improve stability, we should apply a biodegradable and biocompatible SiO2 layer on the surface of MNPs. We also employ a polymer called chitosan that has been cross-linked with glutaraldehyde to enhance the surface characteristics of MNPs.

## Materials and methods

Sigma Aldrich (MO, USA) supplied the following materials: sodium sulfite (Na_2_SO_3_), sodium hydroxide (NaOH, 99%), ammonia solution (25 wt%), hydrochloric acid; 35–37% (w/w), sodium acetate (C_2_H_3_NaO_2_), tetraethyl orthosilicate (TEOS), glutaraldehyde (25 wt%), ethanol (96%), and vancomycin hydrochloride. Merck supplied blood agar, Mueller Hinton (MH) broth, polyethylene glycol (PEG-400), medium molecular weight chitosan (MMWC, deacetylation degree of 75–85%), and nisin from Lactococcus lactis 2.5% (balancing sodium chloride) (Darmstadt, Germany). Penicillin, streptomycin, fetal bovine serum (FBS), and Dulbecco's modified Eagle's medium (DMEM) were acquired from Gibco, USA. We have also used *S. aureus* ATCC 33591 (MRSA, ARSA) and* S. aureus* ATCC 25923 (MSSA) donated by the Hamadan University of Medical Sciences, Hamadan, Iran.

### *Preparation of the Fe*_*3*_*O*_*4*_* MNPs*

The monodispersed Fe_3_O_4_ MNPs were synthesized based on the chemical co-precipitation method as already described with some modifications [41].

### *Synthesis of the Fe*_*3*_*O*_*4*_*@SiO*_*2*_* core/shell nanocomposites*

The Stöber method, as described in the literature, was used to synthesis the Fe_3_O_4_/SiO_2_ MNPs. [42] with some modifications. Generally, 2.00 g of the as-prepared MNPs were soaked into a mixed solvent containing 160 mL of ethanol, 40 mL of deionized water, and 25%v/v concentrated ammonia solution (4 mL), under an ultrasound irradiation treatment for 30 min. Thereafter, under vigorous stirring, tetraethyl orthosilicate (TEOS, 2 mL) was sequentially poured into the resultant suspension and constantly stirred for a further 12 h at 25 °C. The yielded Fe_3_O_4_@SiO_2_ nanocomposites were thoroughly collected by an external magnet and were dried in an oven at 60 °C for 24 h, after being washed by repeated cycles of distilled water and ethanol to eliminate the unreacted particles.

### *Synthesis of the CS-coated Fe*_*3*_*O*_*4*_*@SiO*_*2*_* nanocomposites*

Firstly, 0.5 g of the CS was dispersed in a 50 mL solution of (2%w/w) acetic acid under mechanical stirring at 50 °C for 20 min. Next, 50 mL of the Fe_3_O_4_@SiO_2_ core/shell nanoparticles solution was introduced into the above CS aqueous solution with continuous stirring at 50 °C. The resulting solution was carefully mixed under an ultrasound vibration with the frequency of 20 kHz for 20 min, and then vigorously stirred for further 3 h at 50 °C. The Fe_3_O_4_@SiO_2_@CS nanocomposites were further modified via cross-linking procedure with the glutaraldehyde reagent (2%) at 40 °C for 2 h. Finally, the precipitated nanostructures were rinsed several times with DI water and ethanol (water: ethanol 50:50) to removal the excess remaining uncoated CS and were then dried under vacuum at 60 °C for 6 h [43].

### Preparation of calibration curve of vancomycin

Using double serial dilutions, standard vancomycin solutions were generated at concentrations ranging from 1.25 to 0.001 mg/mL. A UV–Vis spectrophotometer was used to read the optical density (OD) of all solutions at λ_max_ = 283 nm. Vancomycin-loaded magnetic nanoparticles' loading capacity and release efficiency were estimated using a linear equation.

### *Modification of Fe*_*3*_*O*_*4*_*@SiO*_*2*_*@CS nanocomposites with vancomycin*

The vancomycin-loaded magnetic nanostructures were achieved according to the efficient synthesis approach described by Cevher et al. [44]. The Fe_3_O_4_@SiO_2_@CS nanocomposites (VNP1-VNP3) loaded with vancomycin were produced using varying polymer/drug ratios (w/w) of 1:1, 2:1, and 3:1. In summary, Fe_3_O_4_@SiO_2_@CS nanocomposites weighing 10 mg, 20 mg, and 30 mg were individually dispersed into 12.0 mL of phosphate buffered saline (PBS, pH 7.4) using ultrasonic vibration at 30 W for 15 min in a 20 mL beaker containing 10 mg, 10 mg, and 10 mg of dissolved vancomycin: drug ratios of 1:1 (VNP_1_), 2:1 (VNP_2_) and 3:1 (VNP_3_). Subsequently, each mixture was then emulsified by supplying gentle shaking of the containers at 120 rpm for 48 h at 25 ± 0.1 °C (Orbitek shaker incubator) to avoid aggregation and keep an emulsion stable. 10 mg, 20 mg, and 30 mg Fe_3_O_4_@SiO_2_@CS nanocomposites without vancomycin were concomitantly analyzed as the negative controls. Then, the samples were decanted and washed several times with distilled water and were then dried in a vacuum oven.

### Drug content determination

The UV–Vis spectrophotometer at λ_max_ = 283 nm was applied to determine the vancomycin loading capacity onto the Fe_3_O_4_@SiO_2_@CS nanocomposites. Briefly, 10, 20, and 30 mg of the vancomycin-loaded Fe_3_O_4_@SiO_2_@CS nanocomposites were separately dispersed into 12.0 mL PBS (pH 7.4), after mechanical stirring for 24 h at room temperature, the suspension centrifuged (4000 rpm, 10 min), and filtered through a 0.2 µm filter (spartan syringe filter). Vancomycin-loading content onto the nanocomposites was determined by quantifying the UV absorption spectra of the supernatant by a UV-spectrophotometer at the wavelength of 283 nm in comparison with the corresponding control solutions which were made with the same method by assaying the clear supernatant of the blank Fe_3_O_4_@SiO_2_@CS nanocomposites without antibiotic. All vancomycin release data are presented as the mean of the three measurements.

### In vitro* release experiments*

At 37 °C in PBS (pH 7.4), the in vitro drug release profile was evaluated. Vancomycin-loaded Fe_3_O_4_@SiO_2_@CS nanocomposites weighing about 10, 20, and 30 mg each were individually suspended in the relevant buffer and placed into a dialysis bag (molecular weight cut-off 12,000–14,000 Da). The dialysis bag was then submerged in a specific volume of the dissolving media (100 mL, pH 7.4). After that, the suspension was continuously stirred (125 rpm for 24 h) at 37 °C. A UV–Vis spectrophotometer was used to measure the drug concentration at 283 nm after 2 mL of the aliquots were taken out of the external buffer at the designated intervals (every 1 h) and filtered through a 0.45 µm Millipore Millex-HN filter. The aliquots were then replaced with the same volume of fresh dissolution medium. Three separate drug release trials were carried out, and average results were collected.

### Preparation of calibration curve of nisin

The Bradford reagent, which is based on the development of a compound between the vivid blue dye G and the protein in the solution, was used to calculate the concentration of protein in the solution. The protein–dye combination has a maximum absorbance of 595 nm. Because of the Bradford reagent's exceptional sensitivity, protein molecules can be found in our study at concentrations as low as 0.02 mg/mL. The linear concentration of the nisin molecule used as a reference protein with ranged concentrations from 62.5 to 2000 µg/mL. Finally, the calibration curve of nisin was depicted (R^2^ = 0.998) [45].

### *Preparation of the Nisin-grafted Fe*_*3*_*O*_*4*_*@SiO*_*2*_*@CS nanocomposites*

In order to covalently attach nisin to the Fe_3_O_4_@SiO_2_@CS nanocomposites, 50 μL of 25% v/v glutaraldehyde was added dropwise to 5 mL of an aqueous solution of the nanoparticles (2000 µg/mL Fe_3_O_4_@SiO_2_@CS in sodium acetate buffer, 50 mM, pH 5.5). The resulting mixture was then shaken at a speed of 120 rpm for one hour. The samples were then collected by twice washing them in the acetate buffer solution and centrifuging at a speed of 12,000 rpm for 20 min. Next, a pre-measured quantity of nisin was gradually added to the suspension of NPs. The reaction was allowed to proceed with shaking at 80 rpm overnight. Also, a free Fe_3_O_4_@SiO_2_@CS solution (without bacteriocin) was also prepared as the control [46].

#### Evaluation of the covalent binding content of nisin onto the Fe_3_O_4_@SiO_2_@CS nanocomposites

After centrifuging at 2000×*g* and 4 °C for 15 min to separate the uncoated peptides from the nanoparticles, the nisin-modified nanocomposites were recovered using an ultrafiltration process (Amicon, Ultracel-100K, 100-kDa cut off). Utilizing the Bradford protein test and a UV–Vis micro-spectrophotometer (synergy HTX, Bio Tek Corp, USA) set to 595 nm, the remaining nisin content in the outer tube was determined. Following at least three iterations of each experiment and the computation of average absorbance readings, the final amount of loaded nisin was determined using the formula below [47, 48]:

$$\mathrm{Nisin\,loading\, content }\left(\mathrm{\%}\right)=\frac{\mathrm{Total\, amount\, of\, nisin}-\mathrm{Free\, amount\, of\, nisin\, in\, supernatant\, solution}}{\mathrm{Total\, amount\, of\, nanoparticles}}\times 100.$$

### Characterization methods of prepared nanocomposites

A D8 ADVANCE X-ray diffractometer (Bruker, Germany) under CuKα radiation at a voltage of 40 kV and a current of up to 30 mA was used to obtain the X-ray diffraction (XRD) patterns of the Fe_3_O_4_, Fe_3_O_4_@SiO_2_@CS-VANCO, and Fe_3_O_4_@SiO_2_@CS-NISIN composite nanoparticles in order to examine the crystal structure and level of purity of the materials. Using a field emission scanning electronic microscope (FE-SEM, TSCAN, Czech Republic), the morphological characteristics of the Fe_3_O_4_ and Fe_3_O_4_@SiO_2_ core/shell nanoparticles were seen and analyzed. Using KBr pellets at room temperature, Fourier transform infrared (FTIR) spectroscopy (Bruker Alpha spectrometer) was used to determine the chemical structure of the materials and characterize the various functional groups grafted onto the nanocomposites. With the use of a 7410 vibrating sample magnetometer (VSM, Quantum, USA), the magnetic properties were discovered. A Zeta-sizer Nano-ZS (Malvern, UK) was used to assess the zeta potential and the hydrodynamic diameter (Dh).

### In vitro antibacterial activity of the antimicrobials

#### Determination of minimum inhibitory concentration (MIC)

We monitored the antibacterial effect of the Fe_3_O_4_@SiO_2_ MNPs, vancomycin, the Fe_3_O_4_@SiO_2_@CS-VANCO, nisin, and the Fe_3_O_4_@SiO_2_@CS-NISIN nanocomposites against different *S. aureus* strains, namely MRSA*,* MSSA*,* and ARSA through the serial microdilution method in the MHB, as recommended by NCLSI [49]. The MIC determinations for each of the antimicrobial agents were carried out using 96-well microtiter plates in triplicates. The negative controls contained only sterile, un-inoculated broth, and the inoculated broth was considered as the positive control.

#### Determination of minimum bactericidal concentration (MBC)

Following a period of 24-h incubation, 100 μL aliquots of each test sample with no visible bacterial growth were inoculated onto TSA agar plates and incubated at 37 °C for 24 h, thereafter plates were tested for bacterial viability by observing the microbial growth, after which the bacterial viability of the plates was tested by observing the microbial growth. When a majority of the bacterial inoculum (99.99%) was eradicated at the lowest antimicrobial concentration, it is considered as the MBC endpoint.

#### MTT cytotoxic assay

The cytotoxicity effects of Fe_3_O_4_, Fe_3_O_4_@SiO_2_, Fe_3_O_4_@SiO_2_@CS, nisin, Fe_3_O_4_@SiO_2_@CS-NISIN, vancomycin, and Fe_3_O_4_@SiO_2_@CS-VANCO nanocomposites on the L-929 cell line were assessed using the MTT [3-(4,5-dimethylthiazol-2-yl)-2,5-diphenyl tetrazolium bromide, (Merck, Germany)] assay. In summary, 200 μL of DMEM medium (DNA BioTech, Iran) supplemented with 10% FBS (fetal bovine serum, Invitrogen, USA) and 1% penicillin–streptomycin (100 U/mL penicillin, and 100 μg/mL streptomycin solution, Sigma, USA) was used to plat the cells at a density of 1 × 10^4^ cells/mL in each well. After 24 h of incubation at 37 °C under 5% CO_2_ to 80% confluence, the medium was removed and they were treated with various concentrations of 125, 250, 500, 1000, 1500, 2000, and 4000 μg/mL Fe_3_O_4_, Fe_3_O_4_@SiO_2_, and Fe_3_O_4_@SiO_2_@CS nanocomposites, 12, 24, 48, 96, 192, 384, and 768 μg/mL free nisin, and Fe_3_O_4_@SiO_2_@CS-NISIN nanocomposites, 1, 2, 4, 8, 16, and 32 μg/mL free vancomycin, and Fe_3_O_4_@SiO_2_@CS-VANCO nanocomposites for 24 h at 37 °C. Subsequently, the cells underwent two complete washes in 100 µL of PBS to eliminate any remaining polymers and medicines. Then, 200 μL of fresh DMEM without FBS was added to each well, and 10 µL of the MTT stock solution (0.5 mg/mL in PBS) was added. The wells were then incubated for an additional 4 h at 37 °C and 5% CO_2_. Subsequently, 100 µL of dimethyl sulfoxide (DMSO) was added to the medium solution in order to dissolve the purple formazan crystals that had developed in the cells. The wells were gently shaken for 15 min on an orbital shaker. The absorbance of the wells was measured at λ_max_ = 570 nm using a Biorad H1 M microplate reader to determine the cell viability. The viability of cells after treatment was calculated in comparison to that of the untreated cells considering the absorbance value of the positive control group (100% alive).

### Animal studies

Mice with full-thickness wounds were used to evaluate the in vivo antibacterial activity of Fe_3_O_4_@SiO_2_@CS, Fe_3_O_4_@SiO_2_@CS-NISIN, and Fe_3_O_4_@SiO_2_@CS-VANCO nanocomposites. The National Committee for Laboratory Animal Use and Care's recommendations were followed throughout all animal procedures [50]. Animal protocols were approved by the ethics committee of the Hamadan University of Medical Sciences (No: IRUMSHA. REC. 1398.574), and done according to the ARRIVE criteria. Male NMRI mice (6–8 weeks old) were used as the excision models in the wound healing research. They were acquired from the breeding facility of Hamadan University of Medical Sciences. For a week, each mouse was housed separately in ventilated cages with free access to food and water and a regular light and dark cycle of 12 h. Following two weeks of acclimation, the animals were intraperitoneally injected with xylazine (20 mg/kg, Woerden, Netherlands) and ketamine (40 mg/kg).

Next, a 7 mm full-thickness incision was made using a biopsy punch, and 100 µL of a 3 × 10^8^ concentration of bacterial solution was injected subcutaneously into the area that was visible. The mice were split up into five treatment groups, each with eight mice; group I treated with free vancomycin, group II received the Fe_3_O_4_@SiO_2_@CS nanocomposites; group III treated with the vancomycin-conjugated Fe_3_O_4_@SiO_2_@CS nanocomposites, group IV was treated with free nisin, and finally, group V received the nisin-conjugated Fe_3_O_4_@SiO_2_@CS nanocomposites. The administration of the antimicrobials-conjugated composites was conducted once daily, while the free agents were inoculated into the target sites twice daily in dosing regimens. 30 min later, the wounds were covered with a bandage to maintain uniformity, to allow wound healing. The mice were subsequently anesthetized and sacrificed, briefly, high doses of ketamine and xylazine used for the easy death of mice after performing experiments. Then, the skin sections were homogenized, the bacterial counts were determined by serial dilution at 0, 4, 9, and 14 days post-injury.

### Statistical analysis

To examine any significant differences (P-value < 0.05) between the groups under study, the analysis of variance (ANOVA) and Tukey test were performed. SPSS statistics software version 20.0 was used to do the statistical analysis of the findings. Each test was run three times, and a P-value of less than 0.05 indicated an acceptable degree of significance for the variations in the mean values.

## Results

### Characterization

#### FE-SEM studies

**Fig. 1:**
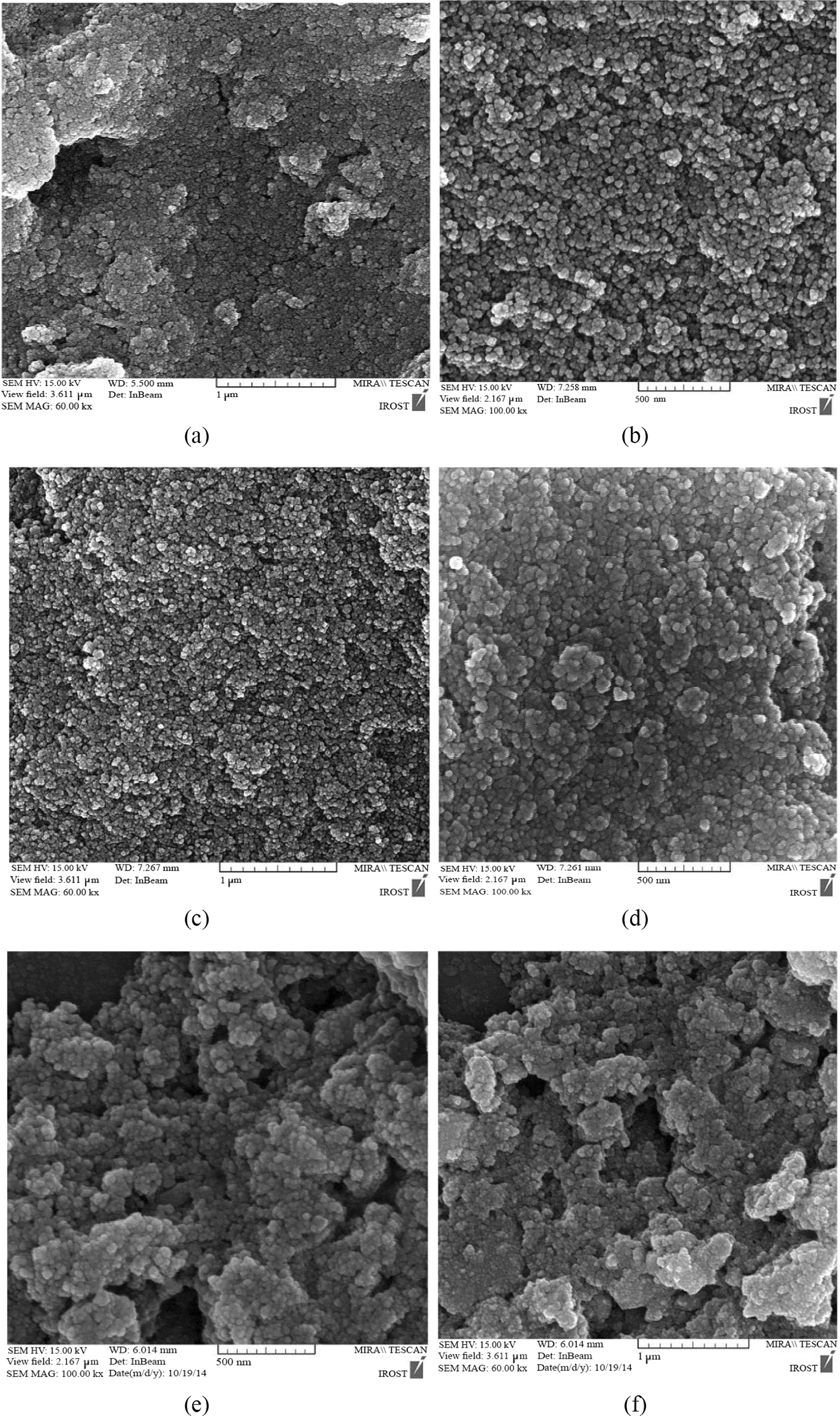
The corresponding FE-SEM images of Fe_3_O_4_ MNPs (**a**, **b**), Fe_3_O_4_@SiO_2_@CS-VANCO (**c**, **d**), and Fe_3_O_4_@SiO_2_@CS-NISIN nanocomposites (**e**, **f**), at different magnifications of (**a**, **c**, **e** 60,000 nm, and **b**, **d**, **f** 100,000 nm)

**Fig. 2:**
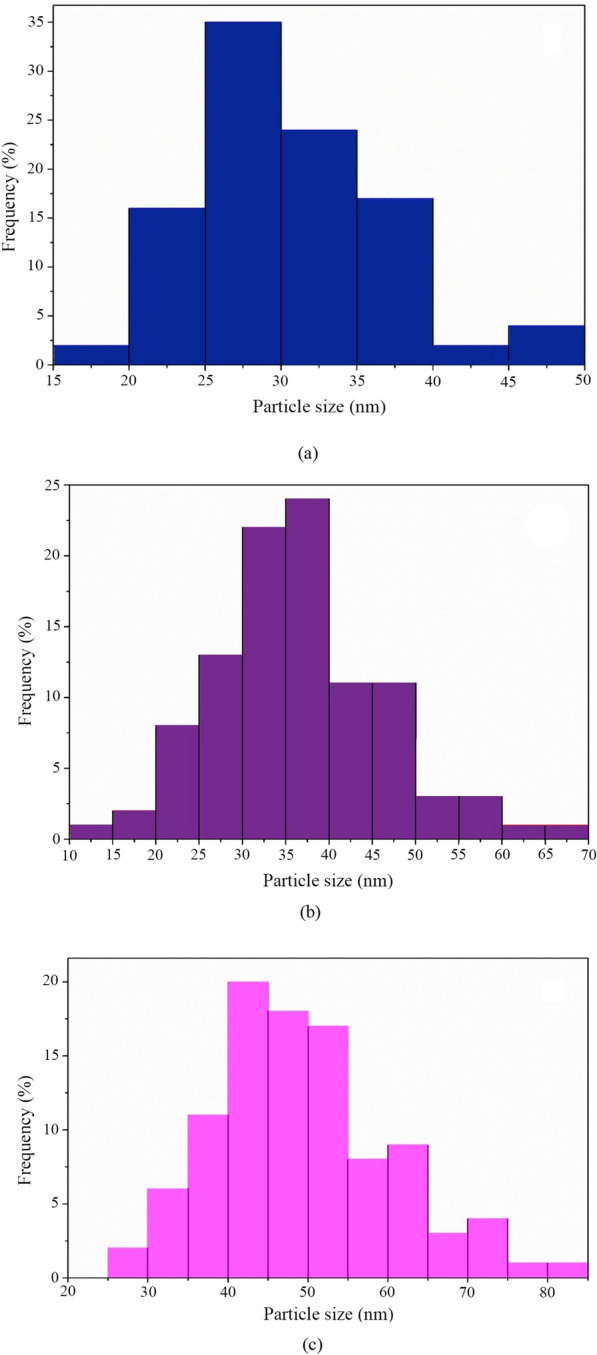
Particle size distribution histogram of Fe_3_O_4_ (**a**), Fe_3_O_4_@SiO_2_@CS-VANCO (**b**), and Fe_3_O_4_@SiO_2_@CS-NISIN nanocomposites (**c**)

Particle size and microstructure of the samples were assessed using FE-SEM analysis, and Fig. 1 displays micrographs of each sample at various magnifications. As can be seen in Fig. 1, in all the samples, the quasi-spherical nanoparticles are visible with different size ranges. To determine the particle size of the samples, a total number of 100 particles were measured from each sample using Image J software and the histograms of the particle size distribution of these measurements are shown in Fig. 2. According to the histograms shown in Fig. 2, 25% of the measured particles in the Fe_3_O_4_ sample had diameters between 25 and 30 nm. The histogram of the Fe_3_O_4_@SiO_2_@ CS-VANCO sample showed the most particles in the 35–40 nm size range, and the histogram of Fe_3_O_4_@SiO_2_@CS-NISIN sample had the greatest number of particles in the size range of 40 to 45 nm by 24%, and 20%, respectively. The statistical data obtained from these measurements are reported in the Table 1. Table 2 clearly shows that, in comparison to Fe_3_O_4_, which had an average particle size of 28.55 nm, the Fe3O4@SiO2@CS-NISIN and Fe3O4@SiO2@CS-VANCO composites had higher particle diameters with average particle sizes of around 47.25 and 34.30 nm, respectively. The presence of organic substances on the MNPs' surfaces could be the cause of this. Furthermore, it is clear that the particle size distribution standard deviations for the Fe_3_O_4_@SiO_2_@CS-NISIN, Fe_3_O_4_@SiO_2_@CS-VANCO, and Fe3O4 samples were 11.14, 9.78, and 6.4 nm, respectively. In other words, the nanocomposites decorated with vancomycin and nisin compared to the Fe_3_O_4_ MNPs, exhibited more deviation from the average particle size values.

**Table 1:** Statistical results of size distribution histograms of as-prepared nanomaterials

Sample	Number of the measured particles	Average particle size (nm)	Standard deviation (nm)	The smallest measured particle (nm)	The measured particle with medium size (nm)	The largest measured particle (nm)
Fe_3_O_4_	100	28.55	6.40	17.43	29.07	47.90
Fe_3_O_4_@SiO_2_@CS-VANCO	100	34.30	9.78	14.03	35.45	65.10
Fe_3_O_4_@SiO_2_@CS-NISIN	100	47.25	11.14	24.43	48.85	82.18

*CS* chitosan, *VANCO* vancomycin

**Table 2:** Actual drug content of vancomycin- functionalized nanoparticles

Polymer:drug ratio	VNP_1_	VNP_2_	VNP_3_
Actual drug loading %	45.52	35.68	24.4

#### FTIR studies

**Fig. 3:**
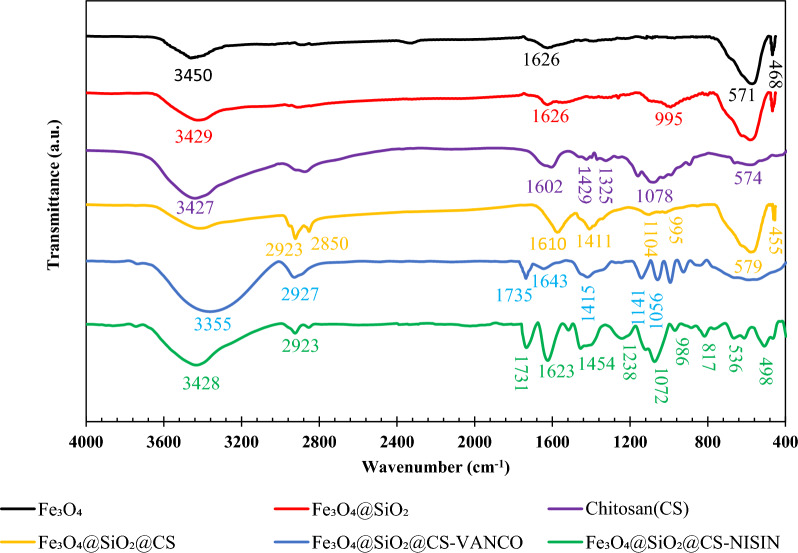
FTIR spectrum of the Fe_3_O_4_, Fe_3_O_4_@SiO_2_, Chitosan (CS), Fe_3_O_4_@SiO_2_@CS, Fe_3_O_4_@SiO_2_@CS-VANCO, and Fe_3_O_4_@SiO_2_@CS-NISIN nanocomposites

Figure 3 presents the FT-IR spectra of (a) Fe_3_O_4_, (b) Fe_3_O_4_@SiO_2_, (c) Fe_3_O_4_@SiO_2_@CS, (d) Fe_3_O_4_@SiO_2_@CS-NISIN, and (e) Fe_3_O_4_@SiO_2_@CS-VANCO nanocomposites. The typical absorption peak centered at 468–571 cm^−1^ in the FT-IR spectra of Fe_3_O_4_ corresponded to the O–Fe–O or Fe–O stretching linked to the magnetite phase of the nanoparticles. The O–H group's bending vibration can be used to explain the adsorption peak at 1626 cm^−1^. The O–H stretching vibration of the water molecules adsorbed on the MNPs' surface is likewise linked to a large peak at 3459 cm^−1^ [51, 52]. Fe_3_O_4_@SiO_2_ nanostructures' spectra show that an absorption band about 995 cm^−1^ was associated with the asymmetric Si–O or Si–OH stretching vibration, indicating that a silica layer had successfully formed on the surface of Fe_3_O_4_ MNPs [53]. The stretching and bending vibrations of the O–H groups of the water molecules in the structure are connected to the absorption bands at 1626 and 3429 cm^−1^, respectively. The spectra of Fe_3_O_4_@SiO_2_@CS demonstrate that the bands at 455, 579, and 455 cm^−1^ correspond to the O–Fe or O–Fe–O stretching mode, while the band at 995 cm^−1^ is caused by the Si–O or Si–OH stretching vibrations. The distinctive bands at 1411 cm-1 are assigned to the stretching vibrations of C–H, verifying the –CH and –CH_2_ groups of CS, while the vibrational band at 1104 cm^−1^ corresponds to the C–O and C–N stretching vibrations, indicating the coating of CS on the surface of Fe_3_O_4_@SiO_2_ NPs. The O–H bending vibrations of H_2_O molecules are linked to an absorption band around 1610 cm^−1^, which also corresponds to the N–H stretching vibrations of the –NH_2_ functional group (the increase in the width of this region compared to the spectrum associated with Fe_3_O_4_@SiO_2_ indicates the presence of the –NH_2_ functional group). The absorption bands in the regions 2850 cm^−1^, and 2923 cm^−1^, arising from the C-H stretching vibrations in the CS structure. In the IR spectra of Fe_3_O_4_@SiO_2_@CS-VANCO, the absorption bands located at 1056 to 1141 cm^−1^ represent the C–O, and C–N stretching vibrations of CS (it is worth noting that the existence of two peaks in this region compared to a nanocomposite with a single band indicates the presence of an additional compound, vancomycin, on the CS-coated magnetic nanocomposites). The stretching vibrations of the C-H in the range of 1415 cm^−1^ are considered to be the –CH and –CH_2_ groups of CS, and also the aromatic C–C (Ar C–C) of vancomycin. The band centered at 1643 cm^−1^ is related to the stretching vibrations of the H–O-H of water molecules and also to the stretching vibrations of the N–H bond belonging to the –NH_2_ functional group. The broad peak at this region when compared to the Fe_3_O_4_@SiO_2_@CS nanocomposite indicates the existence of the NH_2_ group of vancomycin and the successful conjugation of vancomycin molecules onto the surface of nanocomposites. The appearing absorption band at 1735 cm^−1^ is associated with a bond C=O of vancomycin, indicating the presence of vancomycin molecules in the sample structure. The characteristic absorption band located at 2927 cm^−1^ is related to the stretching vibrations of the C–H group of CS, and the stretching vibrations of the –OH groups of water around 3355 cm^−1^ attributed to the presence of H_2_O in Fe_3_O_4_ and the incidence of such an extended spectral peak can be due to the overlapping of the -OH groups occurring in the structure of CS and vancomycin. Regarding the spectra of Fe_3_O_4_@SiO_2_@CS-NISIN, the broad bands appeared at 498 cm^−1^ to 817 cm^−1^, corresponding to the stretching vibrations of Fe–O or O–Fe–O in Fe_3_O_4_ and to the C–H structural skeleton of nisin. The band at 986 cm^−1^ is related to the vibrations of Si–OH or Si–O. The broad absorption band concentrated around 1072 to 1238 cm^−1^ is attributable to the C–O, and C–N stretching vibrations of CS. The stretching vibrations of C-H, which occurred at 1454 cm^−1^, can be assigned to the –CH_3_ and –CH groups of CS, and also to the aromatic C–C bond (Ar C–C) of nisin. The stretching vibrations of the N–H bond of the -NH2 functional group and the H–O–H bond bending mode of water molecules are linked to the absorption band at 1623 cm^−1^. The presence of nisin in the sample is indicated by the band detected at 1731 cm^−1^, which is the result of nisin's C=O bond vibrations. The stretching vibrations of the C-H bond in the CS and nisin structures are indicated by the absorption peak at 2923 cm^−1.^ The stretching vibrations of the O–H group of H2O molecules absorbed into Fe_3_O_4_ are indicated by the absorption band at 3428 cm^−1^, which also demonstrates an overlap of the O–H functional groups present in the CS and nisin structures.

#### VSM studies

**Fig. 4:**
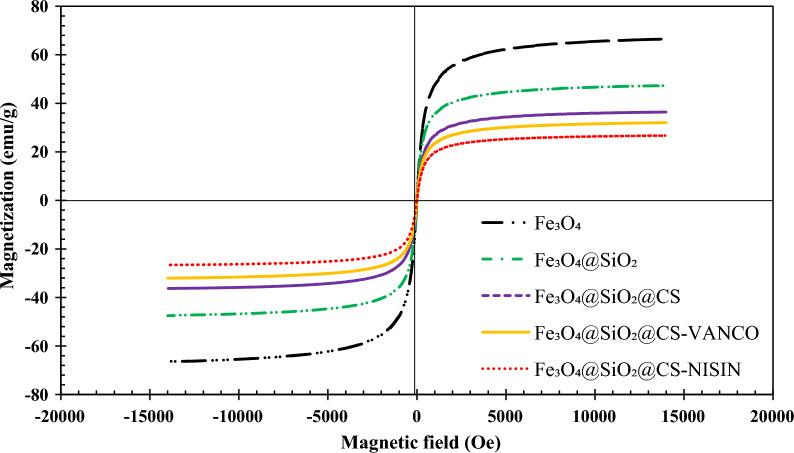
Magnetization curves of Fe_3_O_4_, Fe_3_O_4_@SiO_2_, Fe_3_O_4_@SiO_2_@CS, Fe_3_O_4_@SiO_2_@CS-VANCO, and Fe_3_O_4_@SiO_2_@CS-NISIN nanocomposites

Figure 4 shows the magnetization curves of the nanostructures, which were used to study the magnetic behavior of the suggested nanomaterials using VSM systems. The NPs' superparamagnetic behavior is indicated by the lack of a hysteresis loop, coercivity, and zero remanence at ambient temperature. The saturation magnetization (Ms) of Fe_3_O_4_, Fe_3_O_4_@SiO_2_, Fe_3_O_4_@SiO_2_@CS, Fe_3_O_4_@SiO_2_@CS-VANCO, and Fe_3_O_4_@SiO_2_@CS-NISIN found to be 66.5, 47.3, 36.4, 32.3, and 26.7 emu/g, respectively. Obviously, after the surface functionalization, the modified Fe_3_O_4_@SiO_2_@CS-VANCO, and Fe_3_O_4_@SiO_2_@CS-NISIN composites presented a significant reduction in the Ms value compared to Fe_3_O_4_. The decreased Ms could be attributed to the silanization, the polymeric shell surrounding the magnetite cores, and the displacement of peptide and antibiotic molecules in the nanocomposites, leading to a considerable reduction in the entire magnetic moments.

#### XRD studies

**Fig. 5:**
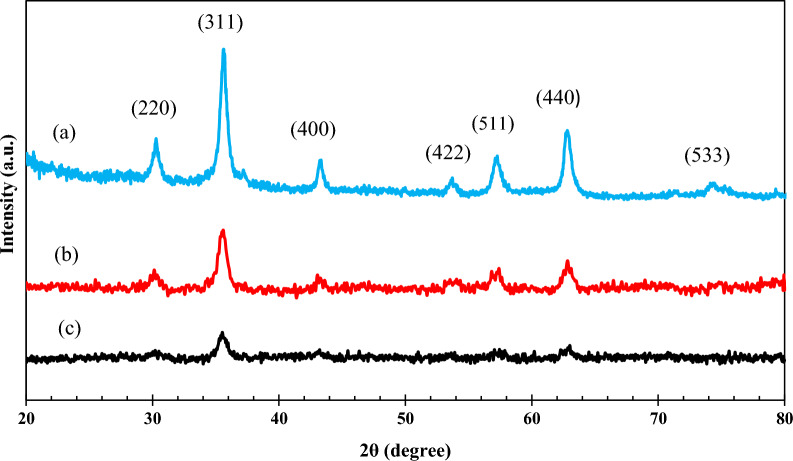
The XRD patterns of Fe_3_O_4_ (**a**), Fe_3_O_4_@SiO_2_@CS-VANCO (**b**), and Fe_3_O_4_@SiO_2_@CS-NISIN (c)

The Highscore plus X’Pert software was used to identify the crystalline phases. Figure 5 clearly shows that in all the three samples the peaks are located at the same angles and differ only in intensity, which means that the crystalline structure is the same and the crystallinity of the structure is different in the three samples. When the peaks of these diffraction patterns were compared with the reference diffraction patterns using the mentioned software, it was found that these samples are based on Fe_3_O_4_ with the reference code JCPDS No. 00-075-0449. Accordingly, all three specimens have a cubic crystal structure and a space group Fm-3 m. In this structure, the diffraction planes (220), (311), (400), (442), (511), (440), and (533) are at 30.3°, 35.7°, 43.4°, 53.9°, 57.3°, 62.8°, and 74.6°, respectively. It can be seen that the peak intensities in the Fe_3_O_4_ nanoparticles are higher than those of Fe_3_O_4_@SiO_2_@CS-NISIN, and Fe_3_O_4_@SiO_2_@CS-VANCO nanocomposites. In fact, the diffraction pattern appears completely crystalline because there are no organic compounds (which are amorphous materials) on the surface of the nanoparticles. When the surface of the NPs is modified with the organic compounds, the intensity of the peaks is reduced, which indicates a decrease in the crystallinity of these compounds. The Scherer equation (Eq. 2) has been used to find the crystalline size of the NPs:

$${\text{D}}=\frac{K\lambda }{\beta {\text{cos}}\left(\theta \right)}$$

In this case, λ is the X-ray wavelength (1.54°A), D is the crystalline size, K is the shape factor, β is the line broadening at half the maximum intensity (FWHM), and θ is the peak location. The Scherer equation is used to determine the crystalline size value given the values of cos (θ) and FWHM, as well as the constant values of λ (1.54°A) and k (0.9). The crystalline size values were obtained to be 54.8, 22.34, and 34.77 nm with Fe_3_O_4_, Fe_3_O_4_@SiO_2_@CS-VANCO, and Fe_3_O_4_@SiO_2_@CS-NISIN nanostructures, respectively. The crystalline size of samples containing organic compounds may have decreased as a result of these compounds sealing the crystalline planes and inhibiting the formation of crystalline materials during the MNP nucleation and growth processes.

#### DLS studies

**Fig. 6:**
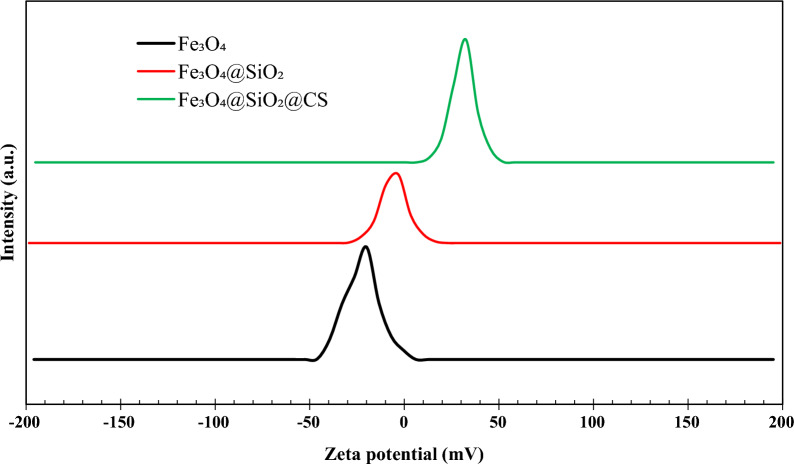
The plots of zeta potential of the magnetic nanomaterials

**Fig. 7:**
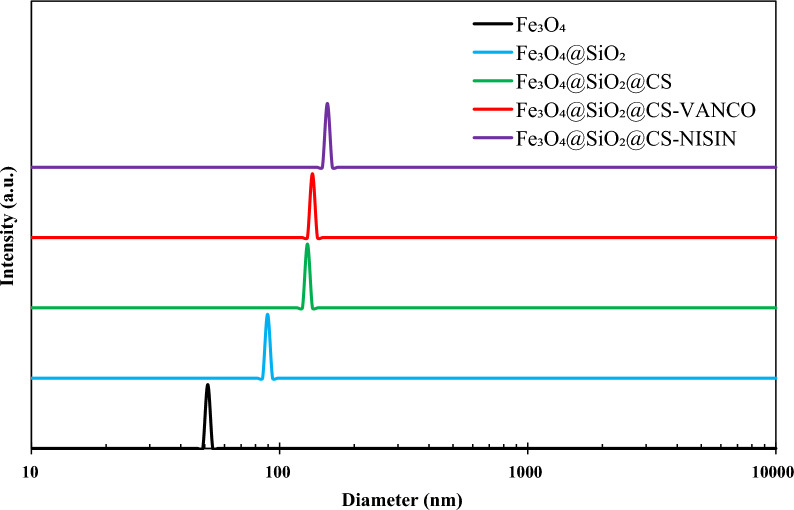
DLS plots of the as-synthesized magnetic nanomaterials

One key measure of colloid stability is the surface electric charge (zeta potential) of the dispersed particles in a colloidal media [54]. According to popular belief, colloidal systems are less stable when the zeta potential absolute value is lower, and an essentially stable suspension is indicated by values above 30 mV [55]. The zeta potential plots of the samples are shown in Fig. 6, in which the zeta potential values of Fe_3_O_4_, Fe_3_O_4_@SiO_2_, and Fe_3_O_4_@SiO_2_@CS were about − 14.25 mV, − 7.19 mV, and 31.74 mV, respectively. The results of the zeta potential values suggest that only the Fe_3_O_4_@SiO_2_@CS had an absolute zeta potential value above 30 mV among the under-experimented samples. Therefore, it can be concluded that Fe_3_O_4_@SiO_2_@CS nanocomposites represents a stable suspension. The hydrodynamic diameter (D_h_) of all the compounds was measured by the dynamic light scattering (DLS) method. According to Fig. 7, the hydrodynamic particles diameter of the Fe_3_O_4_, Fe_3_O_4_@SiO_2_, Fe_3_O_4_@SiO_2_@CS, Fe_3_O_4_@SiO_2_@CS-VANCO, and Fe_3_O_4_@SiO_2_@CS-NISIN nanocomposites was estimated to be 51.4 nm, 93.7 nm, 129.5 nm, 135.7 nm, and 155.8 nm, respectively. The results revealed that the hydrodynamic sizes of Fe_3_O_4_, and Fe_3_O_4_@SiO_2_ nanostructures were calculated to be larger than those estimated from SEM due to the hydration layers in the aqueous solutions. After the surface functionalization with CS, vancomycin, and nisin, the dispersion difficulties of the nanostructures lead to a gradual increase in the nanocomposites size. These findings indicate that vancomycin and nisin have been successfully conjugated to a CS polymer grafted with the silica shell.

### Preparation of calibration curve of vancomycin

**Fig. 8:**
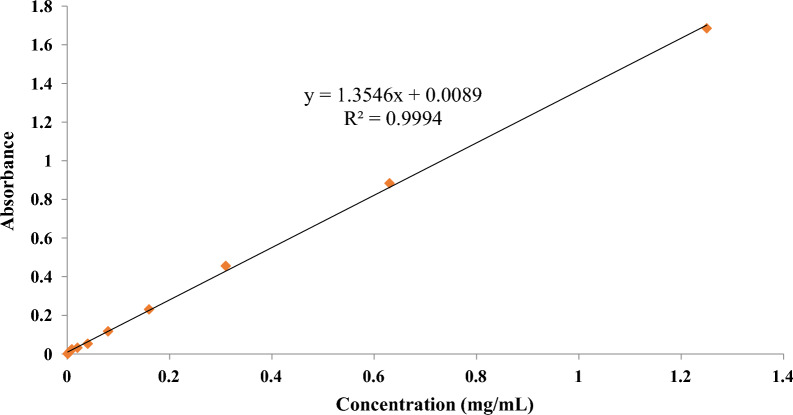
The standard calibration curve of vancomycin

A standard calibration curve was derived from 11 different concentrations of vancomycin with a correlation coefficient of 0.999 for the drugs released into the medium to be quantified (Fig. 8).

### Actual drug content of Fe_3_O_4_@SiO_2_@CS-VANCO nanocomposites

The vancomycin-loaded Fe_3_O_4_@SiO_2_@CS nanocomposites were successfully prepared by changing the nanocomposite-to-drug ratios (three formulations of nanocomposite and drug ratios, VNP_1_-VNP_3_). The drug loading percentage of each mixture was separately calculated and the results are presented in Table 2. A high loading efficiency (over 50%) was achieved at a polymer: drug ratio of 1:1.

### In vitro release profiles of vancomycin-functionalized Fe_3_O_4_@SiO_2_@CS nanocomposites

**Fig. 9:**
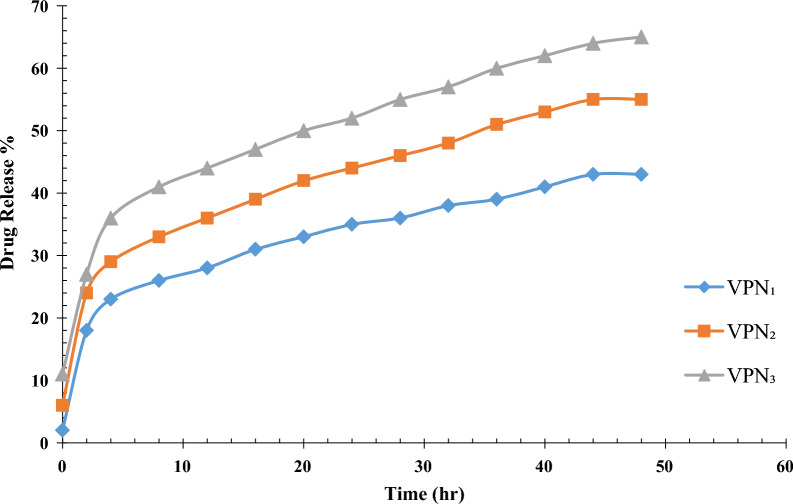
The in vitro vancomycin release profile of VNP_1_, VNP_2_, and VNP_3_ nanocomposites in PBS (pH 7.4) at 37 °C

Figure 9 shows vancomycin release profiles from Fe_3_O_4_@SiO_2_@CS nanocomposites in PBS (pH 7.4) at 37 °C. The VNP_1_ and VNP_3_ had the slowest and the highest dissolution rates, respectively. In vitro, the drug release rate from the composites decreased as the polymer/drug ratio decreased as the following arrangement: VNP_3_ > VNP_2_ > VNP_1_. 43% of total drug were released from VNP_1_ over 48 h, whereas 55%, and 65% of the drugs were released from VNP_2_ and VNP_3_, respectively, over the same period. We selected VNP_1_ to investigate its antimicrobial properties for in vivo studies because the slow release of the drug can reduce the time between administration and the occurrence of adverse events, thus improving patient compliance [56].

### The covalent binding content of nisin onto the nanocomposites

The covalently bonded amount of nisin was calculated using an ultrafiltration technique and measuring the absorbance of supernatants at 595 nm by an UV–Vis spectrophotometer. The nisin loading content based on the linear equation (y = 0.0001x + 0.0176, R^2^ = 998) was calculated to be between 73.2 and 87.2%, which was described by the generation of a calibration curve for nisin with sodium acetate buffer (50 mM, pH 5.5).

### Cytotoxicity assessment of the therapeutic agents

SF1 displays the outcomes of the MTT cell-proliferation experiment used to analyze the viability of the L-929 cell line. The cytotoxicity of the produced compounds was assessed after a period of 24 h incubation. These findings imply that Fe_3_O_4_ MNPs did not significantly inhibit the growth of L-929 cells, even up to a high exposure concentration (4000 μg/mL), at which the cell viability decreased to 63%. Furthermore, the existence of a silica shell on the surface of Fe_3_O_4_ MNPs could lead to a reduced cell density in the L-929 cells by around 3–7%, which corresponded to the determined concentrations for MIC and MBC after 24 h of treatment with the nano-conjugated Fe_3_O_4_@SiO_2_ in comparison with the naked Fe_3_O_4_. The Fe_3_O_4_@SiO_2_ nanostructures exhibited a good degree cyto-compatibility with a cell viability percentage of > 80 at a concentration corresponding to the MIC value, and even at a high concentration of 2000 μg/mL (eightfold MIC), calculated to be 63%. It clearly demonstrated that the decoration of core/shell nanocomposites with CS could considerably improve the cell proliferation in comparison with the bare Fe_3_O_4_, and Fe_3_O_4_@SiO_2_ MNPs (P-value = 0.007). The Fe_3_O_4_@SiO_2_@CS nanostructures did not exert any significant effect on the reduction of the total number of viable cells at a concentration corresponding to a 125 µg/mL MIC dose with a cell viability of 100%. Furthermore, it was observed that as the concentration of CS-modified MNPs increased, the cell viability continued to be remarkable after 24 h of incubation at a 4000 µg/mL concentration of the Fe_3_O_4_@SiO_2_@CS nanomaterials (viable cells > 70%). The information presented in this work showed that the viability of mouse fibroblast cells was largely unaffected by free nisin. These results also showed that even at high nisin concentrations (12–768 µg/mL), the nisin-functionalized Fe_3_O_4_@SiO_2_@CS nanocomposites could not inhibit the proliferation of cells. The free vancomycin, and vancomycin-grafted Fe_3_O_4_@SiO_2_@CS nanostructures had almost no inhibitory effect on the viability of these cell lines at the range of concentrations tested (1–32 µg/mL), even following exposure to the highest concentration (32 µg/mL), the percent of viable cells was calculated to be 97%, for free vancomycin, and 100% for the Fe_3_O_4_@SiO_2_@CS-VANCO nanoconjugates.

### In vitro antibacterial efficacy of antimicrobials

According to the findings presented in Table 3, all the compounds at sufficient quantities have the capacity to impede the bacterial growth in the following order: Fe_3_O_4_@SiO_2_@CS-VANCO > Vancomycin > Nisin > Fe_3_O_4_@SiO_2_@CS-NISIN > Fe_3_O_4_@SiO_2_@CS > Fe_3_O_4_@SiO_2_. The MIC value for Fe_3_O_4_@SiO_2_ MNPs was found in the range of 250 µg/mL against MRSA, MSSA, and ARSA strains, the MBC values were determined to be 500, and 250 µg/mL against MRSA, and MSSA/ARSA strains, respectively. After treatment with the CS-modified Fe_3_O_4_@SiO_2_ particles, both the MIC, and MBC values were reduced by two-fold as compared to the naked MNPs. As listed in the Table 3, the MIC, and MBC values of Fe_3_O_4_@SiO_2_@CS nanocomposites against MRSA determined to be around 125, and 250 µg/mL, respectively, and against the both strains MSSA, and ARSA about 125 µg/mL. The bactericidal performance of Fe_3_O_4_@SiO_2_ MNPs against MRSA was observed at a higher concentration (500 µg/mL) than that of against MSSA/ARSA (250 µg/mL), and when exposed to the CS-coated MNPs, these values decreased up to twofold against the both *S. aureus* strains. In accordance with other works, it can be concluded that the CS-modified MNPs can achieve a superior antibacterial performance as compared with bare MNPs [57]. The improved inhibitory activity of vancomycin-functionalized nanocomposites indicated that they are more effective antibacterial agents against *S. aureus* strains compared to free vancomycin, as the MIC and MBC toxicity concentrations were found to be twofold lower in comparison with the use of free vancomycin. The vancomycin-conjugated Fe_3_O_4_@SiO_2_@CS composites had the potential to reduce the MIC and MBC doses from 1.84, 1.62, and 1.46 µg/mL to 0.92, 0.81, and 0.73 µg/mL against MRSA, ARSA, and MSSA strains, respectively. In this study, nisin also showed an excellent antibacterial effect at the MIC and MBC concentrations of about 12–18 µg/mL, while the covalent conjugation of nisin on the surface of nanocomposites resulted in a less toxic effect than free nisin with the MIC and MBC values of about 48–52 µg/mL against all strains.

**Table 3:** Minimum inhibitory concentration (MIC; µg) and minimum bactericidal concentration (MBC; µg) for six antibacterial agents on three strains of *Staphylococcus aureus*

*S. aureus* species	Antibacterial agents (μg/mL)											
	Fe_3_O_4_@SiO_2_		Fe_3_O_4_@SiO_2_@CS		Nisin		Fe_3_O_4_@SiO_2_@CS NISIN-		Vancomycin		Fe_3_O_4_@SiO_2_@CS-VANCO	
	MIC	MBC	MIC	MBC	MIC	MBC	MIC	MBC	MIC	MBC	MIC	MBC
MRSA	250	500	125	250	18	18	52	52	1.84	1.84	0.92	0.92
MSSA	250	250	125	125	12	12	48	48	1.46	1.46	0.73	0.73
ARSA	250	250	125	125	12	18	48	52	1.62	1.62	0.81	0.81

*S. aureus Staphylococcus aureus,* *MRSA and MSSA* methicillin-resistant and -susceptible *S. aureus*, *ARSA* ampicillin-resistant *S. aureus*, *MIC* minimum inhibitory concentration, *MBC* minimum bactericidal concentration, *CS* chitosan, *VANCO* vancomycin

### Animal studies

Therapeutic efficacy of Fe_3_O_4_@SiO_2_@CS nanocomposites containing a peptide and an antibiotic against MRSA was monitored in murine models of the infected surgical wounds (NMRI). Analysis of the bacterial counts in the surgical site infections on a spectrum of the five treatment groups was recorded over different time points (0, 4, 9, and 14 days). All the compounds exhibited the sufficient antibacterial performance in the following order: Fe_3_O_4_@SiO_2_@CS-VANCO > Vancomycin > Nisin > Fe_3_O_4_@SiO_2_@CS-NISIN > Fe_3_O_4_@SiO_2_-CS nanocomposites in agreement with in vitro antibacterial efficacy (Table 4). The results showed that treatment with Fe_3_O_4_@SiO_2_@CS-VANCO nanocomposites resulted in a considerable reduction in the bacterial colony counts of the surgical incisions compared to the other materials after 9 and 14 days of the initial exposure to nanostructures (P-value = 0.002). Groups treated with the un-modified Fe_3_O_4_@SiO_2_@CS nanocomposites showed a lower reduction in the bacterial burden as compared with the other therapeutic agents at the end of 4th, 9th, and 14th days (P-value < 0.05), followed by the nanostructures containing nisin. The nisin-decorated Fe_3_O_4_@SiO_2_@CS nanocomposites were capable of exhibiting a better antibacterial efficacy than the exposure to Fe_3_O_4_@SiO_2_@CS alone (P-value = 0.001). The superior efficiency of free nisin to inhibit the bacterial growth compared to nisin conjugated-materials during 14 days post-treatments was demonstrated. The bacterial count of wounds that had received the free nisin dressings amounted to 10^6^ (5.2 ± 1.7), 10^4^ (6.2 ± 0.9), and 210 ± 23 CFU/mL, while wounds treated with nisin-grafted Fe_3_O_4_@SiO_2_@CS nanocomposites had the total bacterial burden of 10^7^ (4.3 ± 0.4), 10^5^ (1.4 ± 0.3), and 280 ± 23 CFU/mL, over the 4th, 9th, and 14th of the treatment days, respectively. As displayed in SF2, when the mice were treated with Fe_3_O_4_@SiO_2_@CS-NISIN and Fe_3_O_4_@SiO_2_@CS-VANCO nanocomposites, the wound healing process was more accelerated than those exposed to vancomycin and free nisin because of the synergistic effects of chitosan with the other therapeutic agents in the reduction of the bacterial CFU and a decreased diameter of the wound scar area.

**Table 4:** Bacterial counts (CFU/g) of a mouse skin abrasion lesion on the 4th, 9th, and 14th days after treatment

Day	MRSA	Free vancomycin	Fe_3_O_4_@SiO_2_@CS	Fe_3_O_4_@SiO_2_@CS-VANCO	Free nisin	Fe_3_O_4_@SiO_2_@CS-NISIN
0	3 × 10^7^	5.4 × 10^7^	5.5 × 10^7^	5.4 × 10^7^	5.4 × 10^7^	5.4 × 10^7^
4	3 × 10^7^	4.6 × 10^6^ ± 1.8 × 10^6^	5.4 × 10^7^ ± 1.7 × 10^7^	3.6 × 10^5^ ± 2.2 × 10^5^	5.2 × 10^6^ ± 1.7 × 10^6^	4.3 × 10^7^ ± 0.4 × 10^7^
9	3 × 10^7^	5.7 × 10^4^ ± 0.8 × 10^4^	3.7 × 10^5^ ± 1.4 × 10^5^	6 × 10^3^ ± 1.5 × 10^3^	6.2 × 10^4^ ± 0.9 × 10^4^	1.4 × 10^5^ ± 0.3 × 10^5^
14	3 × 10^7^	178 ± 15	315 ± 51	108 ± 12	210 ± 23	280 ± 23

*MRSA* methicillin-resistant *Staphylococcus aureus*, *CS* chitosan, *VANCO* vancomycin

## Discussion

Recently, drug resistance among *S. aureus* strains is rapidly expanding in worldwide and the conventional therapeutic approaches have challenged in treatment of infections caused by them [58]. The development of novel strategy for effectively eradicating resistant staphylococcal infections, particularly those related by MRSA, is urgently necessitated [59]. In the present study, the anti-MRSA/ARSA activities of Fe_3_O_4_@SiO_2_, Fe_3_O_4_@SiO_2_@CS-VANCO, Fe_3_O_4_@SiO_2_@CS-NISIN composites were investigated and the high potential of MNPs as an effective antibacterial agent was proven. According to our data, Fe_3_O_4_@SiO_2_ MNPs had the bacteriostatic and bactericidal properties in the MIC/MBC = 250 µg/mL concentration range against the MSSA/ARSA strains and under MIC = 500 µg/mL and MBC = 250 µg/mL against MRSA. Also, we synthesized Fe_3_O_4_@SiO_2_@CS-VANCO composite with a significant anti- MRSA potential, which reduced the MIC and MBC values of free drug. In another study, the MIC and MBC values of zinc oxide NPs against MRSA strains were observed in very low, ranged from 312.5 to 1250 μg/ml, indicating significant antibacterial activity of MNPs [60]. Also, the confirmatory study showed the excellent bacteriostatic activity of silver NPs against *S. aureus* strains, where the MICs for MSSA and MRSA were detected 25 μg/mL and 50 μg/mL, respectively [61]. The bactericidal efficacy of MNPs can be attributed to numerous mechanisms. One of the main mechanisms through which antibacterial agents exert their adverse effects is oxidative damage caused by reactive oxygen species (ROS) radicals, which can damage enzymes in the bacterial antioxidant system (SOD, catalase, and glutathione reductase), proteins, and DNA in bacteria [62, 63]. Park et al. found that the bactericidal property of the silver metals is due to the accumulation of ROS in the bacterial cells [64]. Tran et al. showed that the metal oxide Fe_3_O_4_ and its ROS effectively mediated the inhibition of *S. aureus* [63]. The metal ions have the ability to interact with the active functional groups of proteins, including enzymes, such as mercapto (–SH), amino (–NH), and carboxyl (–COOH) groups, which can affect the structure and the function of enzyme systems [65]. Keenan et al. described a similar process in which ferrous iron (Fe^2+^) can react with the molecular oxygen to produce the hydrogen peroxide, which eventually reacts with Fe^2+^ to produce the hydroxyl radicals that can destroy the biological macromolecules [66]. Furthermore, the direct binding of iron oxide nanoparticles (IONPs) to the bacterial cell wall greatly disrupts the structural integrity and alters the selective permeability of the cell membrane [67, 68]. The direct interaction of IONPs with the cell wall of *S. aureus* was studied by the method of SEM [68]. The IONPs can also penetrate and concentrate in the cytoplasm, triggering the formation of cavities and the rupture of cell walls [69]. In the current study, chitosan coated-magnetic nanostructures exhibited an unrivaled antibacterial way of behaving over Fe_3_O_4_@SiO_2_, implying that the expansion of chitosan onto the MNPs surface could improve the antimicrobial capability of formulated MNPs. A variety of mechanisms can affect the effectiveness of the CS inhibitor, including the interaction of polycationic CS NH_3_^+^ groups with the anionic moieties on the bacterial cell wall, which alters the cell permeability and consequently the infiltration of intracellular constituents and membrane lysis [70, 71]. The binding of hydrolyzed materials to microbial DNA, interfering with mRNA and protein synthesis [72, 73]. The CS can organize in the form of deposited polymer films on the cell surface, blocking nutrients access and oxygen diffusion, thereby suppressing the growing cells of aerobic bacteria [72, 74, 75]. The CS can inhibit microbial growth and toxin production by chelating essential nutrients, metal ions, and trace elements [71, 72]. It has also been demonstrated that the functionalization of IONPs with CS can lead to its enhanced bactericidal activity against *Bacillus subtilis*, and *Escherichia coli,* which is associated with the production of ROS [76]. Ghiass et al. developed Fe_3_O_4_@CS@Ag, and Fe_3_O_4_@CS@Ag@CH_2_COOH nanocomposites which presented the promising antibacterial properties against Gram-negative *Esherichia coli* (*E. coli*), and Gram-positive *S. aureus* with a great cellular viability and biocompatibility on the L-929 cell lines [77]. In a study to investigate the bactericidal activity of different nanocomposites against *S. aureus, Staphylococcus epidermidis (S. epidermidis), B. subtilis*, and MRSA, Esmaeili et al. [43], reported that the surface modification of MnFe_2_O_4_ with CS resulted in a two-fold reduction in the MIC value for MRSA (MIC value of MnFe_2_O_4_ and chi-MnFe_2_O_4_ ≥ 2500, and 1250 mg/mL, respectively), while for other bacteria the suppressive dose of MnFe_2_O_4_ was fourfold higher than that of Ch-MnFe_2_O_4_ (the MIC values of MnFe_2_O_4_, and chi-MnFe_2_O_4_ = 2500, and 625 mg/mL, respectively). Some researchers have shown that nisin affects bacterial growth through a dual-cidal mechanism by targeting the membrane-bound peptidoglycan precursor lipid II, leading to the disruption of cell-wall biosynthesis and the formation of pores in the cell membranes [78]. In addition, nisin can also serve as a trigger for the autolytic process in *Staphylococci* [79]. Nisin and nisin-loaded nanostructures had significant inhibitory effects against the studied strains both in vivo and in vitro. Various assays have proven the potency of antimicrobial peptides (AMPs), including nisin, for treating bacterial infections. Zhao et al. found that synthesized Ag-Nisin NPs had remarkable antimicrobial and anti-biofilm effects targeting a scope of both Gram-negative and -positive bacterial pathogens, which were 2–4 times more efficient than the clinical utilization of ionic silver. Particularly, the as-prepared Ag-nisin NPs displayed a reduced degree of cytotoxicity compared to ionic silver particles towards mammalian cells [80]. In one study, intranasal administration of Nisin-F effectively suppressed the growth of *S. aureus* in the airways of immunodeficient rats [81]. In another study, Staden et al. reported that subcutaneous injection of Nisin-F-loaded brushite bone cement into the dorsal subcutaneous pocket of mice was effective in controlling *S. aureus* Xen 36 infection over a 7 day period [82]. Okuda et al. explored the anti-biofilm potential of nisin to control the biofilm formation of MRSA on medical equipment and found that nisin A relative to the other two bacteriocins including lacticin Q and nukacin ISK-1 had the greatest effectiveness in inhibiting biofilm formation [21]. Heunis et al. announced that nisin-containing nanofibers can serve as effective wound dressings, potentially reducing the bacterial load of *S. aureus*-infected surgical wounds, as well as shortening the healing time of surgical wounds [83]. Unfortunately, not only IONPs can exhibit bacteriostatic and bactericidal behaviors and unique magnetic properties, but also cytotoxicity to certain eukaryotic cell lines. The most commonly proposed mechanism involved in the cytotoxic effects of IONPs is the induction of ROS production, which may lead to an increased lipid peroxidation, decreased antioxidant enzymes, and the promotion of protein aggregation [84–87]. Additionally, the extra concentrations of IONPs can enhance lipid metabolism, disrupt iron homeostasis, and aggravate the impairment of mouse liver function in vivo [88]. The NPs may induce a decrease in the viability of cells with increasing concentration and longer exposure time [89]. The viability of L-929 fibroblast cells was further reduced when the concentration of nanomaterials was increased. The results are presented in Fig. 8, which clearly suggest that the toxic effects of Fe_3_O_4_@SiO_2_@CS composites on the mouse fibroblast cells (L-929) were significantly less than those of the bare MNPs. The lower toxicity of Fe_3_O_4_@SiO_2_@CS nanostructures can be explained by the sustained release of iron ions leading to ROS-mediated cell death, resulting in a significant reduction in the toxicity of cells treated with Fe_3_O_4_@SiO_2_@CS [85, 90]. The higher toxicity of bare IONPs is associated with the greater intracellular release and in situ degradation of iron ions [85]. All the results undoubtedly support the theory that a polycationic CS coating would be an effective strategy to reduce the toxic effects of IONPs. This is in agreement with the results reported by Shukla et al., who showed the reduction of IONPs toxicity following chitosan oligosaccharide coating due to the sustained release of Fe^2+^ ions, causing ROS formation and subsequent cell death [89]. In this regard, Guo et al., demonstrated that the dimercaptosuccinic acid (DMSA)-coated particles can induce less cellular cytotoxicity compared to bare Fe_3_O_4_, and Fe_3_O_4_@SiO_2_, thus preventing direct interaction between the nanomaterials and the human dermal fibroblasts. The DMSA-modified particles had a slightly significant effect on the number of viable cells even under very high dosages and prolonged exposure times. Furthermore, in line with our findings, the coating of a SiO_2_ layer on the MNPs led to a decrease in the cell viability [91]. These findings indicate a good biocompatibility and a low cytotoxicity of NPs, which may provide the suitable nano-vehicles for their application in the biomedical field. At concentrations ranging from 16 to 32 μg/mL, vancomycin caused a greater dose-dependent growth inhibition on L-929 cells than Fe_3_O_4_@SiO_2_@CS-VANCO nanocomposites. It is possible that the controlled release of drug can reduce the cytotoxicity of Fe_3_O_4_@SiO_2_@CS-VANCO nanocomposites [92]. In the last decade, the application of MNPs to overcome bacterial infections has expanded, and their high potency has been proven to successfully eradicating of wound infections [93]. In this regard, the present study approved the extensive antimicrobial properties of MNPs which could be attributed to ROS production, targeting functional groups of metabolites, cell membrane damage, as well as disruption in protein function, electron transfer chains, and repair systems [94–96]. Also, in another study, the outstanding features of silver NPs including chemical stability, catalytic activity, broad-spectrum antibacterial ability, etc. were confirmed, indicating their high potential in treatment of infectious wounds [97]. In addition, the antimicrobial and healing properties of zinc oxide NPs have been proven, which could regenerate damaged skin by controlling infection, re-epithelialization, reducing necrosis, and collagen fiber deposition [98]. Moreover, in the conducted study by Sivaranjani et al., titanium dioxide NPs were known as powerful wound healer agent that could effectively control skin infections caused by both Gram-positive and -negative bacteria [99]. Like mentioned studies, copper NPs exhibited high microbicidal abilities. Also, these NPs could enhance the wound healing process by involving in angiogenesis, collagen formation, and enhancing immunity responses [100]. However, mechanism of actions, affordability, cytotoxicity effects, and synthesis methods, and are among the main factors which ought to be considered for MNPs development in bacterial wound healing [101]. Magnetic-based hemostatic and wound healing systems are currently being developed with the particles decorated with bioactive materials [102]. The efficient antibacterial activity and sufficient biocompatibility offer further opportunities for the treatment of in vivo infectious diseases caused by the pathogens. It can document that the surface modification of IONPs can be an ideal way to improve their antibacterial capacity. In this regard, it is worth noting that the antibacterial effect of free nisin was more potent in comparison with Fe_3_O_4_@SiO_2_@CS-NISIN nanostructures (P-value = 0.001), under in vitro*/*in vivo conditions (Tables 2 and 3), indicating that the antibacterial effects of some of the nisin molecules became inactivated during the conjugation process. The presence of glutaraldehyde molecules on the surface of the nanocomposites may have interfered with the binding of some nisin molecules to the MRSA cell wall, or they may have blocked nisin active sites. Further research can focus on the development of more efficient approaches to nisin conjugating in order to eliminate or minimize the loss of nisin performance. This implies the binding and active sites of nisin remain un-changed during the chemical reactions. Based on our data, the chitosan-coated nanostructures, which exhibited lower inhibitory performance than the other therapeutic agents, were effective in considerably suppressing the growth of strains, so that the MRSA CFUs strains were reduced over the 4th day from 10^7^ (5.4 ± 1.7) and 10^7^ (5.8 ± 2.4) to 315 ± 51 and 360 ± 14 on the 14th day after surgical wounds exposure to these nanostructures, respectively. Moreover, the incorporation of MNPs (Fe_3_O_4_@SiO_2_) into the CS polymers can improve the wound healing performance by enhancing the physical and antibacterial properties of CS [103, 104], and by reducing the cytotoxicity of Fe_3_O_4_@SiO_2_ MNPs incorporated into CS polymers [105, 106]. An example of the ferria-based healing composites was presented in the work of Shabanova et al. in which a number of various functional drugs trapped in the magnetite films allowed for a gradual release and improved healing efficiency with a greatly contraction in the scar size [102]. Besides its own intrinsic biocompatible and non-toxic characteristics, CS also exerts some effectual inhibitory effects and wound healing properties related to its polycationic nature, such as hemostatic, and analgesic properties [107]. Additionally, CS may release a substance that triggers the fibroblast proliferation and the collagen synthesis, all of which contribute to infected site healing and to accelerate in the wound repair process [108]. Some studies have suggested that the CS dressings based on metallic nanoparticles can accelerate the wound closure not only by promoting inflammatory inhibition but also by enhancing collagen accumulation, and angiogenesis [109, 110]. Numerous reports have highlighted the use of CS as a perfect wound dressing material due to its beneficial capacity to acceleration of the wound-closure processes, besides its biocompatibility, biodegradability, antimicrobial, and hemostatic activities [111]. The results revealed that the vancomycin-functionalized nanocomposites had more efficient antibacterial properties compared to the topical exposure to un-modified nanocomposites and free vancomycin on days 4, 9, and 14 after the treatment. Long-term protection of the drugs from natural inhibitors, enzymatic degradation, and other adverse factors has been achieved by chemically conjugating of the therapeutic agents to NPs, which enhances the efficacy of antimicrobials [112]. We propose that vancomycin has a bactericidal effect by destroying the bacterial cell wall, enhancing the capability of nanocomposites to penetrate the bacterial cells and reach their target sites resulting in more potent antibacterial effects compared to the undecorated nanocomposites. Exposure of the bacterial cell wall to nanocomposites and changes in the cell membrane permeability can also potentially contribute to vancomycin in the inhibition of bacterial cells growth. The efficacy of vancomycin in treating *S. aureus* infections was noticeably improved along with the nanoparticle-based strategies at a lower antibiotic dose, due to the controlled release of vancomycin during the treatment process, thereby reducing side effects. In agreement with our work, Zhou et al. demonstrated that vancomycin-functionalized nanostructures had higher antibacterial potency against drug-resistant enterococcal strains than untargeted nanoparticles also the materials were more potent towards *Enterococcus. faecalis* strains in vivo as compared to nanoparticles without vancomycin [113]. In this regard, Hagbani et al. revealed that vancomycin-modified gold nanoparticles (V-GNPs) had an extremely higher efficiency even at low doses relative to free vancomycin [114]. Similarly, Hussain et al. concluded that the vancomycin-carrying cyclic 9-amino acid peptide CARGGLKSC (CARG) nanoparticles had a greater effectiveness to eradicate staphylococcal infections in vivo in the lung tissue of *S. aureus*-infected mice in contrast with similar dosages of unmodified nanoparticles or free vancomycin [115]. Esmaeili et al., indicated that vancomycin-conjugated magnetic nanocomposites resulted in about twofold and fourfold reduction in the MIC values towards Gram-positive and -negative bacteria in comparison to free vancomycin, respectively [43]. In this study, the size of the synthesized MNPs was measured using zeta-sizer and FE-SEM. The comparison of the data obtained from these two methods showed that the nanoparticles' size reported by FE-SEM was more uniformity than which obtained by zeta-sizer, resulting SEM method should be implemented to enhance the reproducibility and sensitivity of the size analysis even more [116]. In addition, data obtained from DLS method demonstrated the uniformity in zeta potentials of synthesized MNPs, indicating that SiO_2_ is a suitable core–shell substrate for these nanocomposites that can be applied to acquire reliable results [117]. In addition, the drug release profile was conducted in triplicate and the average data were taken. We ensured the reproducibility and efficacy of the release profile of Fe3O4@SiO2@CS-VANCO nanocomposites through a control sample containing vancomycin in the free form [118]. This could attribute that the dialysis bag membrane was not a barrier during the release study. Moreover, the MICs and MBCs value of synthesized nanocomposites were examined and similar results were obtained.

in triplicate, confirming the reproducibility of in vitro antimicrobial studies. However, genotype of analyzed bacteria, MNPs' synthesis process, time intervals between analyses, are among the variable conditions that could affect reproducibility of MIC and MBC results [119]. Several limitations of our study included the following: since we had many assays and errors in formulating the suitable nanomaterials, the time required to complete the project was very long. This research involves numerous items of the equipment, and since they were all not centralized at one place, working became extremely more difficult.

## Conclusion

In this paper, Fe_3_O_4_@SiO_2_@CS-VANCO and Fe_3_O_4_@SiO_2_@CS-NISIN MNPs with a high magnetic property (26.7 and 32.3 emu/g, respectively) were synthesized by a two-step facile procedure. Vancomycin and nisin as the potential therapeutic agents were satisfactorily linked to biocompatible and biodegradable superparamagnetic nanocomposites, which are characterized by a sufficient release profile, suitable size, and spherical shape. Vancomycin-loaded Fe_3_O_4_@SiO_2_@CS nanocomposites were successfully prepared and a high loading efficiency (over 50%) was achieved at a polymer: drug ratio of 1:1. Also, the nisin was grafted onto the nanocomposites at around 73.2–87.2%. The data indicated that the magnetic nanocomposites had a controlled dissolution profile in vitro. The comparison of the vancomycin release profiles from Fe_3_O_4_@SiO_2_@CS nanocomposites showed the lowest release profile from the polymer/drug ratio 1:1, which total released drug from this formulation was examined 43% during 48 h. Also, the MIC and MBC concentrations of Fe_3_O_4_@SiO_2_@CS-VANCO against MSSA/ARSA were found to be twofold lower in comparison to free vancomycin, indicating the improved in vitro antibacterial activity of vancomycin-functionalized nanocomposites. In both in vitro*/*in vivo, the inhibitory effects of free nisin had a about fourfold toxicity effect in suppressing the growth of bacterial cells compared to nisin grafted onto the nanocomposites, which may be due to the inactivation of some nisin molecules during the linkage process. Moreover, the MTT assay suggests that exposure to Fe_3_O_4_@SiO_2_@CS-VANCO nanoconjugates had almost no inhibitory effect on the viability of on L-929 fibroblasts cells at the range of concentrations tested (1–32 µg/mL), confirming their great biocompatibility for in vivo applications. Finally, Fe_3_O_4_@SiO_2_@CS-VANCO nanocomposites resulted in a considerable reduction in the bacterial colony counts of the surgical incisions, suggesting an ideal tool to deal with therapeutic challenges caused by wound infections.

### Supplementary Information

Below is the link to the electronic supplementary material.

**Additional file 1.** Representation of the cell viability rate of the L-929 cells after 24 h following exposure with different treatments of (**a**) Fe_3_O_4_, Fe_3_O_4_@SiO_2_, and Fe_3_O_4_@SiO_2_@CS nanocarriers, (**b**) vancomycin, and Fe_3_O_4_@SiO_2_@CS-VANCO nanocomposites, (**c**) nisin and Fe_3_O_4_@SiO_2_@CS-NISIN nanocomposites
**Additional file 2.** Photographs of wound healing process in the groups of MRSA-infected mice exposed to Fe_3_O_4_@SiO_2_@CS, free nisin, Fe_3_O_4_@SiO_2_@CS-NISIN, vancomycin and Fe_3_O_4_@SiO_2_@CS-VANCO nanocomposites during days 0, 4, 9, and 14 post-surgical incision

## Data Availability

The datasets used and/or analyzed during the current study are available from the corresponding author on reasonable request.

## References

[1] Organization WH. Prioritization of pathogens to guide discovery, research and development of new antibiotics for drug-resistant bacterial infections, including tuberculosis. World Health Organization; 2017. Report No.: 9240026436.

[2] O’Neill J. Review on antimicrobial resistance Antimicrobial resistance: tackling a crisis for the health and wealth of nations. 2014;2014(4).

[3] Control CfD, Prevention. Antibiotic resistance threats in the United States, 2019: US Department of Health and Human Services, Centres for Disease Control and…; 2019.

[4] De Oliveira DM, Forde BM, Kidd TJ, Harris PN, Schembri MA, Beatson SA (2020). Antimicrobial resistance in ESKAPE pathogens. Clin Microbiol Rev.

[5] Cieplak T, Soffer N, Sulakvelidze A, Nielsen DS (2018). A bacteriophage cocktail targeting *Escherichia coli* reduces *E. coli* in simulated gut conditions, while preserving a non-targeted representative commensal normal microbiota. Gut microbes..

[6] Wan F, Draz MS, Gu M, Yu W, Ruan Z, Luo Q (2021). Novel strategy to combat antibiotic resistance: a sight into the combination of CRISPR/Cas9 and nanoparticles. Pharmaceutics.

[7] Imran M, Jha SK, Hasan N, Insaf A, Shrestha J, Shrestha J (2022). Overcoming multidrug resistance of antibiotics via nanodelivery systems. Pharmaceutics.

[8] Chiozzi V, Rossi F (2020). Inorganic–organic core/shell nanoparticles: progress and applications. Nanoscale Adv.

[9] Zarrintaj P, Saeb MR, Jafari SH, Mozafari M (2020). Application of compatibilized polymer blends in biomedical fields. Compatibilization of polymer blends.

[10] Chenthamara D, Subramaniam S, Ramakrishnan SG, Krishnaswamy S, Essa MM, Lin F-H (2019). Therapeutic efficacy of nanoparticles and routes of administration. Biomater Res.

[11] Yao Y, Zhou Y, Liu L, Xu Y, Chen Q, Wang Y (2020). Nanoparticle-based drug delivery in cancer therapy and its role in overcoming drug resistance. Front Mol Biosci.

[12] Turner CT, McInnes SJ, Voelcker NH, Cowin AJ (2015). Therapeutic potential of inorganic nanoparticles for the delivery of monoclonal antibodies. J Nanomater..

[13] Dizaj SM, Jafari S, Khosroushahi AY (2014). A sight on the current nanoparticle-based gene delivery vectors. Nanoscale Res Lett.

[14] Poon C, Gallo J, Joo J, Chang T, Bañobre-López M, Chung EJ (2018). Hybrid, metal oxide-peptide amphiphile micelles for molecular magnetic resonance imaging of atherosclerosis. J Nanobiotechnol.

[15] Cord-Landwehr S, Moerschbacher BM (2021). Deciphering the ChitoCode: fungal chitins and chitosans as functional biopolymers. Fungal Biol Biotechnol.

[16] Santos VP, Marques NS, Maia PC, Lima MABd, Franco LdO, Campos-Takaki GMd (2020). Seafood waste as attractive source of chitin and chitosan production and their applications. Int J Mol Sci..

[17] Azmana M, Mahmood S, Hilles AR, Rahman A, Arifin MAB, Ahmed S (2021). A review on chitosan and chitosan-based bionanocomposites: promising material for combatting global issues and its applications. Int J Biol Macromol.

[18] Bibi A, Ibrar M, Shalmani A, Rehan T (2021). A review on recent advances in chitosan applications. Pure Appl Biol (PAB)..

[19] Zou Y, Lee HY, Seo YC, Ahn J (2012). Enhanced antimicrobial activity of nisin-loaded liposomal nanoparticles against foodborne pathogens. J Food Sci.

[20] Brumfitt W, Salton MR, Hamilton-Miller JM (2002). Nisin, alone and combined with peptidoglycan-modulating antibiotics: activity against methicillin-resistant *Staphylococcus aureus* and vancomycin-resistant enterococci. J Antimicrob Chemother.

[21] Okuda K, Zendo T, Sugimoto S, Iwase T, Tajima A, Yamada S (2013). Effects of bacteriocins on methicillin-resistant *Staphylococcus aureus* biofilm. Antimicrob Agents Chemother.

[22] Shin JM, Ateia I, Paulus JR, Liu H, Fenno JC, Rickard AH (2015). Antimicrobial nisin acts against saliva derived multi-species biofilms without cytotoxicity to human oral cells. Front Microbiol.

[23] Prabhu Y, Rao KV, Kumari BS, Kumar VSS, Pavani T (2015). Synthesis of Fe_3_O_4_ nanoparticles and its antibacterial application. Int Nano Lett.

[24] Al-Shabib NA, Husain FM, Ahmed F, Khan RA, Khan MS, Ansari FA (2018). Low temperature synthesis of superparamagnetic iron oxide (Fe_3_O_4_) nanoparticles and their ROS mediated inhibition of biofilm formed by food-associated bacteria. Front Microbiol.

[25] Khan AU, Chen L, Ge G (2021). Recent development for biomedical applications of magnetic nanoparticles. Inorg Chem Commun.

[26] Aisida SO, Akpa PA, Ahmad I, Zhao T-k, Maaza M, Ezema FI (2020). Bio-inspired encapsulation and functionalization of iron oxide nanoparticles for biomedical applications. Eur Polym J.

[27] Karbasaki SS, Bagherzade G, Maleki B, Ghani M (2021). Magnetic Fe_3_O_4_@ SiO_2_ core-shell nanoparticles functionalized with sulfamic acid polyamidoamine (PAMAM) dendrimer for the multicomponent synthesis of polyhydroquinolines and dihydro-1*H*-indeno [1, 2-b] pyridines. Org Prep Proced Int.

[28] Maleki B, Atharifar H, Reiser O, Sabbaghzadeh R (2021). Glutathione-coated magnetic nanoparticles for one-pot synthesis of 1,4-dihydropyridine derivatives. Polycyclic Aromat Compd.

[29] Tarahomi M, Alinezhad H, Maleki B (2019). Immobilizing Pd nanoparticles on the ternary hybrid system of graphene oxide, Fe_3_O_4_ nanoparticles, and PAMAM dendrimer as an efficient support for catalyzing sonogashira coupling reaction. Appl Organomet Chem.

[30] Alinezhad H, Pakzad K, Nasrollahzadeh M (2020). Efficient Sonogashira and A3 coupling reactions catalyzed by biosynthesized magnetic Fe_3_O_4_@ Ni nanoparticles from Euphorbia maculata extract. Appl Organomet Chem.

[31] Ghani M, Zayeri Z, Maleki B (2021). Glutathione-stabilized Fe_3_O_4_ nanoparticles as the sorbent for magnetic solid-phase extraction of diazepam and sertraline from urine samples through quantitation via high-performance liquid chromatography. J Sep Sci.

[32] Hajizadeh F, Maleki B, Zonoz FM, Amiri A (2021). Application of structurally enhanced magnetite cored polyamidoamine dendrimer for knoevenagel condensation. J Iran Chem Soc.

[33] Adibian F, Pourali AR, Maleki B, Baghayeri M, Amiri A (2020). One-pot synthesis of dihydro-1*H*-indeno [1, 2-b] pyridines and tetrahydrobenzo [b] pyran derivatives using a new and efficient nanocomposite catalyst based on *N*-butylsulfonate-functionalized MMWCNTs-D-NH_2_. Polyhedron.

[34] Masoumparast M, Mokhtary M, Kefayati H (2020). Preparation and characterization of polyvinylpyrrolidone/cobalt ferrite functionalized chitosan graphene oxide (CoFe_2_O_4_@ CS@ GO-PVP) nanocomposite. J Polym Eng.

[35] Hu Z-L, Qin S-L, Li Z-H, Xi L-J, Liu W-J, editors. Preparation and surface modification of monodisperse Fe_3_O_4_ nano particles. In: 2nd Annual International Conference on Advanced Material Engineering (AME 2016); 2016: Atlantis Press.

[36] Abbas M, Torati S, Lee C, Rinaldi C, Kim C (2014). Fe_3_O_4_/SiO_2_ core/shell nanocubes: novel coating approach with tunable silica thickness and enhancement in stability and biocompatibility. J Nanomed Nanotechnol.

[37] Deng L, Li Q, Al-Rehili SA, Omar H, Almalik A, Alshamsan A (2016). Hybrid iron oxide–graphene oxide–polysaccharides microcapsule: a micro-matryoshka for on-demand drug release and antitumor therapy in vivo. ACS Appl Mater Interfaces..

[38] Roca A, Carmona D, Miguel-Sancho N, Bomatí-Miguel O, Balas F, Piquer C (2012). Surface functionalization for tailoring the aggregation and magnetic behaviour of silica-coated iron oxide nanostructures. Nanotechnology.

[39] Mokhtary M (2016). Recent advances in catalysts immobilized on magnetic nanoparticles. J Iran Chem Soc.

[40] Sun C, Du K, Fang C, Bhattarai N, Veiseh O, Kievit F (2010). PEG-mediated synthesis of highly dispersive multifunctional superparamagnetic nanoparticles: their physicochemical properties and function in vivo. ACS Nano.

[41] Gao M, Li W, Dong J, Zhang Z, Yang B (2011). Synthesis and characterization of superparamagnetic Fe_3_O_4_@SiO_2_ core–shell composite nanoparticles. World J Condens Matter Phys.

[42] Lei Z, Pang X, Li N, Lin L, Li Y (2009). A novel two-step modifying process for preparation of chitosan-coated Fe_3_O_4_/SiO_2_ microspheres. J Mater Process Technol.

[43] Esmaeili A, Ghobadianpour S (2016). Vancomycin loaded superparamagnetic MnFe_2_O_4_ nanoparticles coated with PEGylated chitosan to enhance antibacterial activity. Int J Pharm.

[44] Cevher E, Orhan Z, Mülazımoğlu L, Şensoy D, Alper M, Yıldız A (2006). Characterization of biodegradable chitosan microspheres containing vancomycin and treatment of experimental osteomyelitis caused by methicillin-resistant *Staphylococcus aureus* with prepared microspheres. Int J Pharm.

[45] Zohri M, Alavidjeh MS, Haririan I, Ardestani MS, Ebrahimi SES, Sani HT (2010). A comparative study between the antibacterial effect of nisin and nisin-loaded chitosan/alginate nanoparticles on the growth of *Staphylococcus aureus* in raw and pasteurized milk samples. Probiotics Antimicrob Proteins.

[46] Behzadi F, Darouie S, Alavi SM, Shariati P, Singh G, Dolatshahi-Pirouz A (2018). Stability and antimicrobial activity of nisin-loaded mesoporous silica nanoparticles: a game-changer in the war against maleficent microbes. J Agric Food Chem.

[47] Davies E, Bevis H, Delves-Broughton J (1997). The use of the bacteriocin, nisin, as a preservative in ricotta-type cheeses to control the food-borne pathogen Listeria monocytogenes. Lett Appl Microbiol.

[48] Oluić-Vuković V (1997). Bradford's distribution: from the classical bibliometric “law” to the more general stochastic models. J Am Soc Informat Sci.

[49] Wayne P. Clinical and Laboratory Standards Institute (CLSI); 2010. Performance standards for antimicrobial susceptibility testing. 2010;20:1–5.

[50] Baumans V, Van Loo P (2013). How to improve housing conditions of laboratory animals: the possibilities of environmental refinement. Vet J.

[51] Saranya T, Parasuraman K, Anbarasu M, Balamurugan K (2015). XRD, FT-IR and SEM study of magnetite (Fe_3_O_4_) nanoparticles prepared by hydrothermal method. Nano Vision.

[52] Aliramaji S, Zamanian A, Sohrabijam Z (2015). Characterization and synthesis of magnetite nanoparticles by innovative sonochemical method. Proc Mater Sci.

[53] Liang Y, Ouyang J, Wang H, Wang W, Chui P, Sun K (2012). Synthesis and characterization of core–shell structured SiO_2_@YVO_4_:Yb^3+^, Er^3+^ microspheres. Appl Surf Sci.

[54] Kasaeian M, Ghasemi E, Ramezanzadeh B, Mahdavian M, Bahlakeh G (2018). A combined experimental and electronic-structure quantum mechanics approach for studying the kinetics and adsorption characteristics of zinc nitrate hexahydrate corrosion inhibitor on the graphene oxide nanosheets. Appl Surf Sci.

[55] Sun D, Kang S, Liu C, Lu Q, Cui L, Hu B (2016). Effect of zeta potential and particle size on the stability of SiO_2_ nanospheres as carrier for ultrasound imaging contrast agents. Int J Electrochem Sci.

[56] Qu J-B, Shao H-H, Jing G-L, Huang F (2013). PEG-chitosan-coated iron oxide nanoparticles with high saturated magnetization as carriers of 10-hydroxycamptothecin: preparation, characterization and cytotoxicity studies. Colloids Surf B.

[57] Nehra P, Chauhan R, Garg N, Verma K (2018). Antibacterial and antifungal activity of chitosan coated iron oxide nanoparticles. Br J Biomed Sci.

[58] Mirza TM, Ali R, Khan HM (2020). Nasal colonization of methicillin resistant *Staphylococcus aureus* in attendants in a tertiary care hospital of Pakistan. J Islamabad Med Dent College.

[59] Hemmati J, Azizi M, Asghari B, Arabestani MR. Multidrug-resistant pathogens in burn wound, prevention, diagnosis, and therapeutic approaches (Conventional Antimicrobials and Nanoparticles). Can J Infect Dis Med Microbiol. 2023;2023. 10.1155/2023/8854311 PMC10386904 37521436

[60] Umamageswari S, Manipriya B, Kalyani M (2018). Evaluation of antibacterial activity of zinc oxide nanoparticles against biofilm producing methicillin resistant *Staphylococcus aureus* (MRSA). Res J Pharm Technol.

[61] Ansari M, Khan H, Khan A (2011). Evaluation of antibacterial activity of silver nanoparticles against MSSA and MSRA on isolates from skin infections. Biol Med.

[62] Mahdy SA, Raheed QJ, Kalaichelvan P (2012). Antimicrobial activity of zero-valent iron nanoparticles. Int J Modern Eng Res.

[63] Tran N, Mir A, Mallik D, Sinha A, Nayar S, Webster TJ (2010). Bactericidal effect of iron oxide nanoparticles on *Staphylococcus aureus*. Int J Nanomed.

[64] Park H-J, Kim JY, Kim J, Lee J-H, Hahn J-S, Gu MB (2009). Silver-ion-mediated reactive oxygen species generation affecting bactericidal activity. Water Res.

[65] Yu J, Zhang W, Li Y, Wang G, Yang L, Jin J (2014). Synthesis, characterization, antimicrobial activity and mechanism of a novel hydroxyapatite whisker/nano zinc oxide biomaterial. Biomed Mater.

[66] Keenan CR, Sedlak DL (2008). Factors affecting the yield of oxidants from the reaction of nanoparticulate zero-valent iron and oxygen. Environ Sci Technol.

[67] Kohanski MA, DePristo MA, Collins JJ (2010). Sublethal antibiotic treatment leads to multidrug resistance via radical-induced mutagenesis. Mol Cell.

[68] Sousa CU, Sequeira D, Kolenko YV, Pinto ISM, Petrovykh DY (2015). Analytical protocols for separation and electron microscopy of nanoparticles interacting with bacterial cells. Anal Chem..

[69] Li Y, Yang D, Wang S, Li C, Xue B, Yang L (2018). The detailed bactericidal process of ferric oxide nanoparticles on *E. coli*. Molecules..

[70] Severino R, Ferrari G, Vu KD, Donsì F, Salmieri S, Lacroix M (2015). Antimicrobial effects of modified chitosan based coating containing nanoemulsion of essential oils, modified atmosphere packaging and gamma irradiation against Escherichia coli O157: H7 and Salmonella Typhimurium on green beans. Food Control.

[71] Chien R-C, Yen M-T, Mau J-L (2016). Antimicrobial and antitumor activities of chitosan from shiitake stipes, compared to commercial chitosan from crab shells. Carbohyd Polym.

[72] Yuan G, Lv H, Tang W, Zhang X, Sun H (2016). Effect of chitosan coating combined with pomegranate peel extract on the quality of Pacific white shrimp during iced storage. Food Control.

[73] Chen S, Wu G, Zeng H (2005). Preparation of high antimicrobial activity thiourea chitosan–Ag+ complex. Carbohyd Polym.

[74] El-Tahlawy KF, El-Bendary MA, Elhendawy AG, Hudson SM (2005). The antimicrobial activity of cotton fabrics treated with different crosslinking agents and chitosan. Carbohyd Polym.

[75] Liu H, Du Y, Yang J, Zhu H (2004). Structural characterization and antimicrobial activity of chitosan/betaine derivative complex. Carbohyd Polym.

[76] Arakha M, Pal S, Samantarrai D, Panigrahi TK, Mallick BC, Pramanik K (2015). Antimicrobial activity of iron oxide nanoparticle upon modulation of nanoparticle-bacteria interface. Sci Rep.

[77] Ghiassi S, Mokhtary M, Sedaghat S, Kefayati H (2019). Preparation, and antibacterial activity of chloroacetic acid immobilized on chitosan coated iron oxide decorated silver nanoparticles as an efficient catalyst for the synthesis of hexahydroquinoline-3-carboxamides. J Inorg Organomet Polym Mater.

[78] Prince A, Sandhu P, Ror P, Dash E, Sharma S, Arakha M (2016). Lipid-II independent antimicrobial mechanism of nisin depends on its crowding and degree of oligomerization. Sci Rep.

[79] Bierbaum G, Sahl H-G (1985). Induction of autolysis of staphylococci by the basic peptide antibiotics Pep 5 and nisin and their influence on the activity of autolytic enzymes. Arch Microbiol.

[80] Zhao X, Kuipers OP (2021). Synthesis of silver-nisin nanoparticles with low cytotoxicity as antimicrobials against biofilm-forming pathogens. Colloids Surf B Biointerfaces.

[81] De Kwaadsteniet M, Doeschate KT, Dicks LM (2009). Nisin F in the treatment of respiratory tract infections caused by *Staphylococcus aureus*. Lett Appl Microbiol.

[82] van Staden AD, Brand AM, Dicks LM (2012). Nisin F-loaded brushite bone cement prevented the growth of *Staphylococcus aureus* in vivo. J Appl Microbiol.

[83] Heunis TD, Smith C, Dicks LM (2013). Evaluation of a nisin-eluting nanofiber scaffold to treat *Staphylococcus aureus*-induced skin infections in mice. Antimicrob Agents Chemother.

[84] Apopa PL, Qian Y, Shao R, Guo NL, Schwegler-Berry D, Pacurari M (2009). Iron oxide nanoparticles induce human microvascular endothelial cell permeability through reactive oxygen species production and microtubule remodeling. Part Fibre Toxicol.

[85] Singh N, Jenkins GJ, Asadi R, Doak SH (2010). Potential toxicity of superparamagnetic iron oxide nanoparticles (SPION). Nano Rev.

[86] Yarjanli Z, Ghaedi K, Esmaeili A, Rahgozar S, Zarrabi A (2017). Iron oxide nanoparticles may damage to the neural tissue through iron accumulation, oxidative stress, and protein aggregation. BMC Neurosci.

[87] Dwivedi S, Siddiqui MA, Farshori NN, Ahamed M, Musarrat J, Al-Khedhairy AA (2014). Synthesis, characterization and toxicological evaluation of iron oxide nanoparticles in human lung alveolar epithelial cells. Colloids Surf B.

[88] Wei Y, Zhao M, Yang F, Mao Y, Xie H, Zhou Q (2016). Iron overload by superparamagnetic iron oxide nanoparticles is a high risk factor in cirrhosis by a systems toxicology assessment. Sci Rep.

[89] Shukla S, Jadaun A, Arora V, Sinha RK, Biyani N, Jain V (2015). In vitro toxicity assessment of chitosan oligosaccharide coated iron oxide nanoparticles. Toxicol Rep.

[90] Malvindi MA, De Matteis V, Galeone A, Brunetti V, Anyfantis GC, Athanassiou A (2014). Toxicity assessment of silica coated iron oxide nanoparticles and biocompatibility improvement by surface engineering. PLoS ONE.

[91] Guo X, Mao F, Wang W, Yang Y, Bai Z (2015). Sulfhydryl-modified Fe_3_O_4_@ SiO_2_ core/shell nanocomposite: synthesis and toxicity assessment in vitro. ACS Appl Mater Interfaces.

[92] Lee JH, Yeo Y (2015). Controlled drug release from pharmaceutical nanocarriers. Chem Eng Sci.

[93] Hemmati J, Chegini Z, Arabestani MR. Niosomal-based drug delivery platforms: a promising therapeutic approach to fight *Staphylococcus aureus* drug resistance. J Nanomater. 2023;2023.

[94] Kadiyala U, Turali-Emre ES, Bahng JH, Kotov NA, Vanepps JS (2018). Unexpected insights into antibacterial activity of zinc oxide nanoparticles against methicillin resistant *Staphylococcus aureus* (MRSA). Nanoscale.

[95] Kedziora A, Speruda M, Krzyzewska E, Rybka J, Lukowiak A, Bugla-Ploskonska G (2018). Similarities and differences between silver ions and silver in nanoforms as antibacterial agents. Int J Mol Sci.

[96] Li H, Gao Y, Li C, Ma G, Shang Y, Sun Y (2016). A comparative study of the antibacterial mechanisms of silver ion and silver nanoparticles by Fourier transform infrared spectroscopy. Vib Spectrosc.

[97] Deepachitra R, Lakshmi RP, Sivaranjani K, Chandra JH, Sastry TP (2015). Nanoparticles embedded biomaterials in wound treatment: a review. J Chem Pharm Sci.

[98] Lansdown AB, Mirastschijski U, Stubbs N, Scanlon E, Ågren MS (2007). Zinc in wound healing: theoretical, experimental, and clinical aspects. Wound Repair Regen.

[99] Sivaranjani V, Philominathan P (2016). Synthesize of titanium dioxide nanoparticles using Moringa oleifera leaves and evaluation of wound healing activity. Wound Med.

[100] Mendes C, Thirupathi A, Corrêa ME, Gu Y, Silveira PC (2022). The use of metallic nanoparticles in wound healing: new perspectives. Int J Mol Sci.

[101] Chandrakala V, Aruna V, Angajala G (2022). Review on metal nanoparticles as nanocarriers: current challenges and perspectives in drug delivery systems. Emergent Mater..

[102] Shabanova EM, Drozdov AS, Fakhardo AF, Dudanov IP, Kovalchuk MS, Vinogradov VV (2018). Thrombin@ Fe_3_O_4_ nanoparticles for use as a hemostatic agent in internal bleeding. Sci Rep.

[103] Granick MS, Baetz NW, Labroo P, Milner S, Li WW, Sopko NA (2019). In vivo expansion and regeneration of full-thickness functional skin with an autologous homologous skin construct: clinical proof of concept for chronic wound healing. Int Wound J.

[104] Salcido R. The cicatrix: the functional stage of wound healing. LWW; 2018. p. 581. 10.1097/01.ASW.0000527793.87923.ba 29240585

[105] Mihai MM, Dima MB, Dima B, Holban AM (2019). Nanomaterials for wound healing and infection control. Materials.

[106] Jatoi AW, Kim IS, Ogasawara H, Ni Q-Q (2019). Characterizations and application of CA/ZnO/AgNP composite nanofibers for sustained antibacterial properties. Mater Sci Eng C.

[107] Xia G, Zhai D, Sun Y, Hou L, Guo X, Wang L (2020). Preparation of a novel asymmetric wettable chitosan-based sponge and its role in promoting chronic wound healing. Carbohyd Polym.

[108] Narita T, Takayama N, Mizuno K, Ohil S, Osawa S (2012). Enhancement of fibroblast adhesion and proliferation on A. oryzae treated chitosan film. Kobunshi Ronbunshu..

[109] Zhaleh S, Hazeri N, Faghihi MR, Maghsoodlou MT (2016). Chitosan: a sustainable, reusable and biodegradable organocatalyst for green synthesis of 1,4-dihydropyridine derivatives under solvent-free condition. Res Chem Intermed.

[110] Croisier F, Jérôme C (2013). Chitosan-based biomaterials for tissue engineering. Eur Polymer J.

[111] Yang X, Liu W, Li N, Wang M, Liang B, Ullah I (2017). Design and development of polysaccharide hemostatic materials and their hemostatic mechanism. Biomater Sci.

[112] Ziv-Polat O, Topaz M, Brosh T, Margel S (2010). Enhancement of incisional wound healing by thrombin conjugated iron oxide nanoparticles. Biomaterials.

[113] Zhou Z, Peng S, Sui M, Chen S, Huang L, Xu H (2018). Multifunctional nanocomplex for surface-enhanced Raman scattering imaging and near-infrared photodynamic antimicrobial therapy of vancomycin-resistant bacteria. Colloids Surf B Biointerfaces.

[114] Hagbani TA, Yadav H, Moin A, Lila ASA, Mehmood K, Alshammari F (2022). Enhancement of vancomycin potential against pathogenic bacterial strains via gold nano-formulations: a nano-antibiotic approach. Materials (Basel)..

[115] Hussain S, Joo J, Kang J, Kim B, Braun GB, She ZG (2018). Antibiotic-loaded nanoparticles targeted to the site of infection enhance antibacterial efficacy. Nat Biomed Eng.

[116] Sadeghi S, Bakhshandeh H, Ahangari Cohan R, Peirovi A, Ehsani P, Norouzian D (2019). Synergistic anti-staphylococcal activity of niosomal recombinant lysostaphin-LL-37. Int J Nanomed..

[117] Roca A, Carmona D, Miguel-Sancho N, Bomatí-Miguel O, Balas F, Piquer C (2012). Surface functionalization for tailoring the aggregation and magnetic behaviour of silica-coated iron oxide nanostructures. Nanotechnology.

[118] Panwar P, Pandey B, Lakhera P, Singh K. Preparation, characterization, and in vitro release study of albendazole-encapsulated nanosize liposomes. Int J Nanomed. 2010:101–8. 10.2147/ijn.s8030 PMC2841488 20309396

[119] Falcón R, Mateo E, Talaya A, Giménez E, Vinuesa V, Clari M (2017). Reproducible measurement of vancomycin MICs within the susceptible range in *Staphylococcus aureus* by a broth microdilution method with a “quasi-continuum” gradient of antibiotic concentrations. Eur J Clin Microbiol Infect Dis.

