# Supplementary material for: Vancomycin and nisin-modified magnetic Fe3O4@SiO2 nanostructures coated with chitosan to enhance antibacterial efficiency against methicillin resistant Staphylococcus aureus (MRSA) infection in a murine superficial wound model
Source: BMC Chem. 2024 Feb 23;18(1):43. doi: 10.1186/s13065-024-01129-y (PMC10893753; doi:10.1186/s13065-024-01129-y)
Supplement: Supplementary file 1 — Additional file 1. Representation of the cell viability rate of the L-929 cells after 24 h following exposure with different treatments of (a) Fe3O4, Fe3O4@SiO2, and Fe3O4@SiO2@CS nanocarriers, (b) vancomycin, and Fe3O4@SiO2@CS-VANCO nanocomposites, (c) nisin and Fe3O4@SiO2@CS-NISIN nanocomposites Additional file 2. Photographs of wound healing process in the groups of MRSA-infected mice exposed to Fe3O4@SiO2@CS, free nisin, Fe3O4@SiO2@CS-NISIN, vancomycin and Fe3O4@SiO2@CS-VANCO nanocomposites during days 0, 4, 9, and 14 post-surgical incision [file 13065_2024_1129_MOESM1_ESM.docx]

**Supplementary File**

(a)

(b)

(c)

SF1: Representation of the cell viability rate of the L-929 cells after 24 hours following exposure with different treatments of (a) Fe_3_O_4_, Fe_3_O_4_@SiO_2_, and Fe_3_O_4_@SiO_2_@CS nanocarriers, (b) vancomycin, and Fe_3_O_4_@SiO_2_@CS-VANCO nanocomposites, (c) nisin and Fe_3_O_4_@SiO_2_@CS-NISIN nanocomposites.

| Fe_3_O_4_@SiO_2_@CS-VANCO | Fe_3_O_4_@SiO_2_@CS-  NISIN | Vancomycin | Nisin | Fe_3_O_4_@SiO_2_@CS |
| --- | --- | --- | --- | --- |

| 0 Day |
| --- |
| 4 Day |
| 9 Day |
| 14 Day |


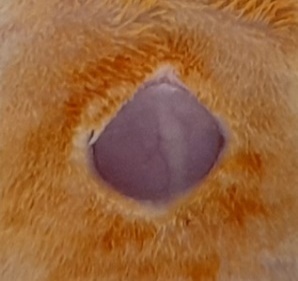
*
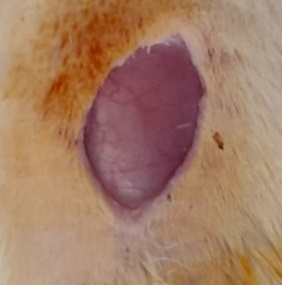
*
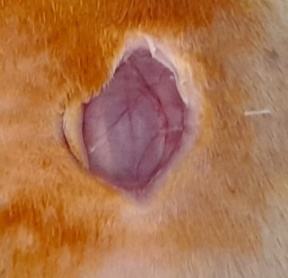


*
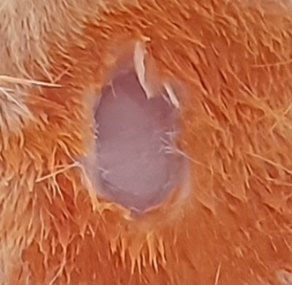
*
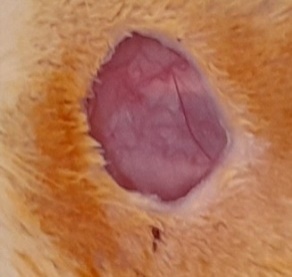


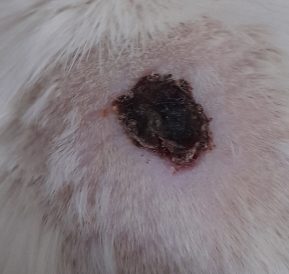
*
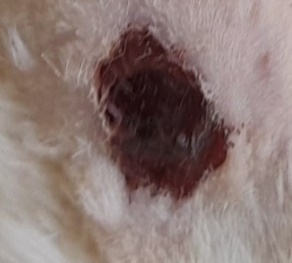
*
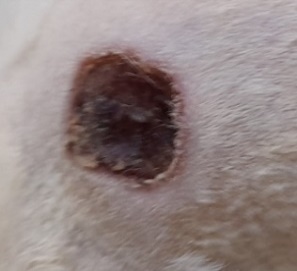

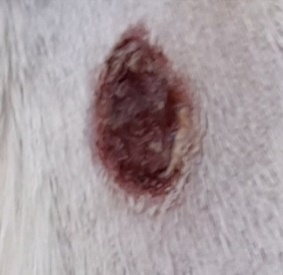
*
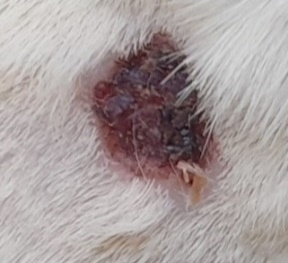
*


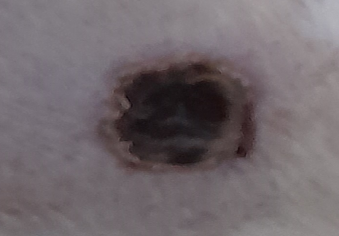
*
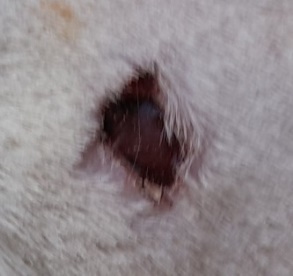

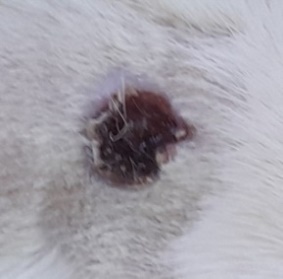

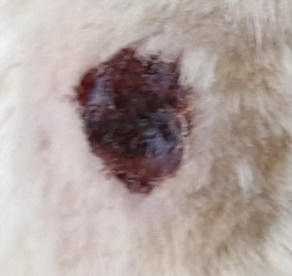

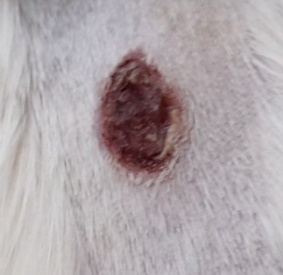
*


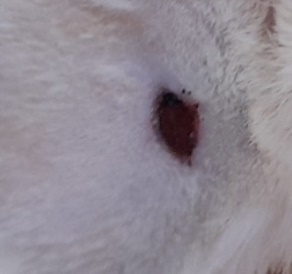
*
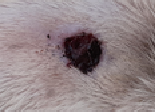

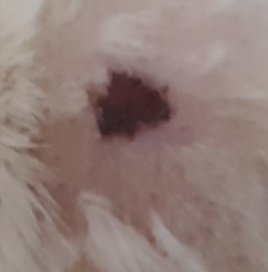

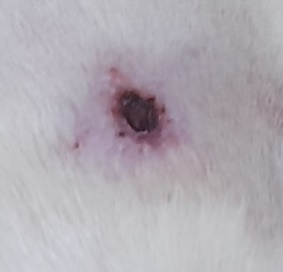

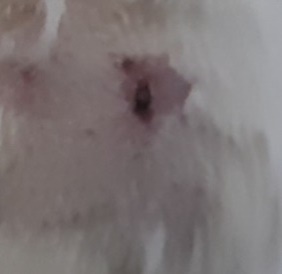
*

SF2: Photographs of wound healing process in the groups of MRSA-infected mice exposed to Fe_3_O_4_@SiO_2_@CS, free nisin, Fe_3_O_4_@SiO_2_@CS-NISIN, vancomycin and Fe_3_O_4_@SiO_2_@CS-VANCO nanocomposites during days 0, 4, 9, and 14 post-surgical incision.
